# High definition transcranial direct current stimulation modulates abnormal neurophysiological activity in post-stroke aphasia

**DOI:** 10.1038/s41598-020-76533-0

**Published:** 2020-11-12

**Authors:** Priyanka P. Shah-Basak, Gayatri Sivaratnam, Selina Teti, Alexander Francois-Nienaber, Maryam Yossofzai, Sabrina Armstrong, Sumiti Nayar, Regina Jokel, Jed Meltzer

**Affiliations:** 1 Rotman Research Institute, Baycrest Health Sciences, Toronto, ON M6A 2E1 Canada; 2 Department of Psychology, University of Toronto, Toronto, ON M5S 1A1 Canada; 3 Department of Speech-Language Pathology, University of Toronto, Toronto, ON M5S 1A1 Canada; 4 Canadian Partnership for Stroke Recovery, Ottawa, ON K1G 5Z3 Canada; 5 Department of Medicine, McMaster University, Hamilton, ON L8S 4L8 Canada; 6 Present Address: Department of Neurology, Medical College of Wisconsin, 8701 W. Watertown Plank Rd, Wauwatosa, Milwaukee, WI 53226 USA

**Keywords:** Diseases of the nervous system, Stroke

## Abstract

Recent findings indicate that measures derived from resting-state magnetoencephalography (rsMEG) are sensitive to cortical dysfunction in post-stroke aphasia. Spectral power and multiscale entropy (MSE) measures show that left-hemispheric areas surrounding the stroke lesion (perilesional) exhibit pathological oscillatory slowing and alterations in signal complexity. In the current study, we tested whether individually-targeted high-definition transcranial direct current stimulation (HD-tDCS) can reduce MEG abnormalities and transiently improve language performance. In eleven chronic aphasia survivors, we devised a method to localize perilesional areas exhibiting peak MSE abnormalities, and subsequently targeted these areas with excitatory/anodal-tDCS, or targeted the contralateral homolog areas with inhibitory/cathodal-tDCS, based on prominent theories of stroke recovery. Pathological MEG slowing in these patients was correlated with aphasia severity. Sentence/phrase repetition accuracy was assessed before and after tDCS. A delayed word reading task was administered inside MEG to assess tDCS-induced neurophysiological changes in relative power and MSE computed on the pre-stimulus and delay task time windows. Results indicated increases in repetition accuracy, decreases in contralateral theta (4–7 Hz) and coarse-scale MSE (slow activity), and increases in perilesional low-gamma (25–50 Hz) and fine-scale MSE (fast activity) after anodal-tDCS, indicating reversal of pathological abnormalities. RsMEG may be a sensitive measure for guiding therapeutic tDCS.

## Introduction

Aphasia is a language disorder typically occurring as a result of a left-hemispheric stroke, affecting 21–38% of the stroke survivors^1^. It is a debilitating condition affecting all modes of verbal communication, including speech and language production, comprehension as well as reading and writing abilities. Lasting impairments negatively impact the social, vocational and emotional quality-of-life in survivors years after their stroke. Outpatient speech-language therapy is available and provides modest benefits^2^. In recent years, there has been a great deal of interest in augmenting therapeutic outcomes with non-invasive brain stimulation, especially using transcranial direct current stimulation (tDCS)^3^.

TDCS is a neuromodulatory, non-invasive brain stimulation technique that operates by exogenously altering neuronal resting membrane potentials and in turn altering cortical excitability, the effects of which outlast the duration of stimulation^4,5^. Depending on the polarity of tDCS electrodes, tDCS has been shown to increase or decrease excitability; anodal-tDCS facilitates and cathodal-tDCS generally inhibits excitability, however recent evidence refutes such linear relationships between polarity and excitability^6^. TDCS as a treatment modality is quite appealing because of its safety and tolerability profile, portability, low-cost, and the ability to easily pair it with behavioral therapies^7^, and thus it is widely used in research settings as a promising neurorehabilitation tool.

In post-stroke aphasia, evidence from dozens of prior original studies and meta-analyses suggests that tDCS can successfully improve language functions, particularly during the chronic phases (more than 3 months after stroke) of recovery^8^. Despite this positive evidence and appeal of tDCS, it is not used routinely in clinical settings to treat aphasia. The clinical applications of tDCS are hampered by the observed mixed efficacy with a few recent reports suggesting no added benefit of tDCS in functional communication^9^. The question of why tDCS seems to benefit some patients and not others, and shows mixed efficacy on the group level, may relate to wide variability in the details of its application and to fundamental uncertainties about its mechanism of action. However, there is general consensus that the placement of electrodes is crucial to determining the effects. Individualized approaches that take structural/lesion and pathological functional characteristics into account can potentially help increase the consistency of tDCS treatment response across patients and thus strengthen its efficacy^10–12^. A recent clinical trial found that *individually targeted* anodal-tDCS, as guided by language task-induced neural activity patterns, was feasible and non-futile, warranting further study for the treatment of aphasia^13^. These findings are promising but currently, there is no consensus on best practices or guidelines that can be used for individualized targeting and for optimizing aphasia recovery using tDCS.

Targeting is especially important as tDCS technology moves toward focal current delivery with high-definition tDCS (HD-tDCS). HD-tDCS replaces the large rubber electrodes with multiple smaller electrodes that are placed strategically on the scalp for focal stimulation^14,15^. As with conventional tDCS, a center-surround configuration of HD-tDCS is safe and well-tolerated^16^, and can either facilitate or inhibit cortical excitability, depending on the polarity of the center electrode relative to the surrounding electrodes. Anodal-tDCS, with anode at the center surrounded by multiple cathodes, is shown to facilitate corticospinal excitability and cathodal-tDCS with the opposite configuration is shown to inhibit excitability^17,18^. The effects of cathodal-tDCS are however, more variable with both HD-tDCS^19^ and conventional-tDCS^6^ applications in motor as well as cognitive domains^20^, and therefore need to be characterized further.

An understanding of the neurophysiological changes induced by tDCS, including HD-tDCS, also remains elusive. Functional magnetic resonance imaging (fMRI) studies in aphasia have found correlational evidence suggesting improved ipsilesional functional connectivity and decreases in overall brain activity after stimulation with conventional-tDCS^21,22^. While these results are critical for our understanding of neural changes in response to tDCS, fMRI as a methodology is limited as it is insensitive to changes in neuronal oscillatory activity occurring in higher temporal frequencies because of its low temporal resolution. In contrast, electrophysiological methods such as magnetoencephalography (MEG) offer higher temporal resolution and may be better equipped to characterize modulatory changes in neuronal activity as a direct consequence of tDCS.

In the current study, we used MEG for: (1) individualized targeting of tDCS, and (2) examining polarity-dependent changes, i.e. after anodal- and cathodal-tDCS, on neuronal oscillatory dynamics in post-stroke aphasia. The individualized selection of targets for tDCS was based on the measurements of multi-scale entropy (MSE), derived from resting-state MEG (rsMEG). We have previously shown that MSE as well as oscillatory or spectral power (the strength of neural oscillations) measures in different frequency bands, both derived from rsMEG, are sensitive indicators of pathological neuronal activity, particularly in areas adjacent to the stroke lesion (perilesional areas). This activity is expressed as slow-wave oscillatory activity and alterations in MSE^23,24^. Specifically, perilesional tissue in chronic stroke consistently exhibit both increased power in the delta (1–4 Hz) and theta (4–7 Hz) frequency bands and reduced power in the beta (15–30 Hz) band, which indicate an overall shift towards lower frequencies. Candidate mechanisms that have been suggested underlying such shifts are subtle neuronal dysfunction, related to factors such as hypoperfusion, disconnection, and selective neuronal loss^24,25^. The increase in lower frequencies after stroke has been associated with cognitive impairments as well as stroke recovery^26–28^, and reductions in the lower frequency power have been linked to improvement of language functions in response to aphasia interventions^28,29^.

Complementary to the spectral power measures, which quantify the oscillatory characteristics of time-varying MEG signals, MSE quantifies the “time-structure of brain activity fluctuations” or temporal patterns in the signals, potentially providing important insights into their nonlinear dynamics^30^. It comprises estimates of sample entropy^31^ across a range of temporal scales^32^. Sample entropy quantifies regularity or predictability by identifying reproducible patterns in a signal over time^30^. Sample entropy and regularity are inversely related: Higher entropy indicates lesser predictability, more complexity or decreased regularity in signals, which is generally associated with healthy processing in the brain. Conversely, lower entropy indicates more predictability (e.g., in signals dominated by periodic oscillations), lower complexity or increased regularity, which is related to dysfunction and is observed in neurological disorders, including traumatic brain injury, tumors, and Alzheimer’s disease^33–36^.

Assessment of sample entropy across multiple time scales as is done with MSE is achieved by successively low-pass filtering and downsampling the signals before computing sample entropy. As a result, an estimate of entropy is available at different time scales, ranging from *fine* (or short) to *coarse* (long) scales, which refer to the time windows used for successive downsampling. Conceptually, coarse-scale entropy has been shown to reflect complexity in lower frequency dynamics, and recent evidence indicates that fine-scale entropy may be sensitive to both high as well as low frequency dynamics^37^. In our prior study in stroke patients with aphasia, we found that perilesional areas exhibit reduced entropy in the fine-scale range and that the same areas also exhibit increased entropy in the coarse-scale range, both compared to MSE from the homolog, contralateral, right hemispheric areas^23,24^. We interpreted these findings in perilesional areas as follows: less complexity in signals (or more predictable signals) with more of the higher frequency content (as in fine scale), and more complexity in signals with low frequency content (as in coarse scale). Together both spectral and MSE measures indicate physiological abnormalities after stroke in perilesional tissue in comparison with the intact hemisphere, which we believe can provide effective ways to localize targets for treatments using tDCS and to potentially restore or normalize function in these areas.

We know that over the course of stroke recovery, i.e. from acute to chronic phases after stroke, increased recruitment of perilesional areas is associated with better language outcomes^38^. The more these areas are involved in the chronic phases, the better the language outcome. Thus, we and others have rationalized that targeting perilesional areas with excitatory or anodal-tDCS could increase functional recruitment of these areas and in turn augment recovery^39^. Alternatively, based on the interhemispheric inhibition account, the suppression of right hemispheric, contralesional activity with cathodal-tDCS could support language recovery^40^. Balanced inhibition between the two hemispheres is disturbed due to the left-hemispheric stroke, enabling contralesional, right-hemispheric areas to become increasingly involved. Such contralesional disinhibition could interfere with the ability of perilesional areas to contribute to language recovery^41–43^. Thus, the model would suggest that suppression of right hemispheric contralesional activity could augment recovery. We investigated how anodal-tDCS of the perilesional areas and cathodal-tDCS, which may be inhibitory, of the homolog contralateral areas affect linguistic performance, and oscillatory and temporal dynamics using spectral power and MSE, respectively.

In the current double-blinded, randomized, sham-controlled, serial HD-tDCS‒MEG study, we assessed the efficacy of rsMEG-guided target localization methods employed with perilesional (left) anodal-tDCS and right homolog cathodal-tDCS in eleven stroke survivors with aphasia. We examined immediate polarity-dependent changes on (1) linguistic performance using a sentence/phrase repetition task and (2) MSE and spectral power measures at the site of stimulation. We hypothesized that repetition performance would improve after both anodal- and cathodal-tDCS and that both would normalize the oscillatory and temporal activity in perilesional areas, compared to sham-tDCS. The extent of these changes would be positively correlated with performance changes. With MEG, we expected reductions in the low frequency power (in delta and/or theta [1–7 Hz]) and increases in higher frequency power (in beta and low-gamma [15–50 Hz]) after both active tDCS conditions compared to sham-tDCS. Additionally, we expected increased fine-scale and reduced coarse-scale entropy, compared to sham-tDCS. Ten age-matched controls were also recruited to further evaluate group differences and pathological activity compared to stroke patients using MEG measures without any stimulation (Supplementary Table 1). Our study provides important insights into polarity-dependent behavioral and neurophysiological changes with individually-targeted HD-tDCS using task-free approaches in post-stroke aphasia.

## Results

**Figure 1:**
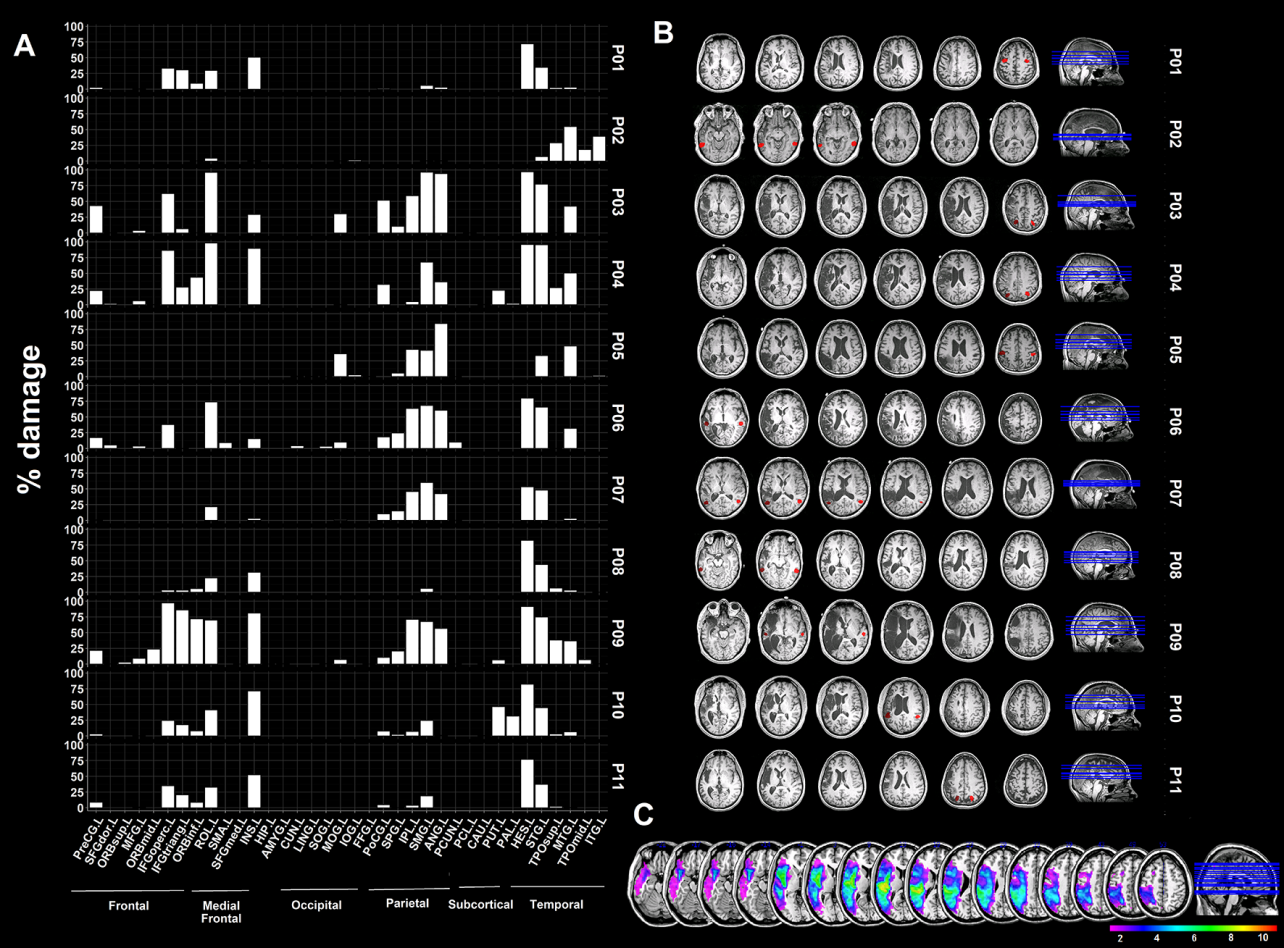
(**A**) Barplots indicating percentage (%) of damage in eleven stroke survivors who completed the study. Manually drawn lesions are summarized by calculating % damage within each of the 90 regions as defined by the automated anatomical labeling atlas (AAL). (**B**) T1 images of individual patients indicating lesion locations, and overlaid stimulation sites represented in red or maroon spheres; note the stimulation sites in the left hemisphere are in perilesional areas, and the stimulation sites in the right hemisphere are mirror/homologs of the left sites. (**C**) Lesion overlap map presenting the distribution of lesions across 11 patients. *PreCG* Precentral gyrus; *SFGdor* Superior frontal gyrus, dorsolateral; *ORBsup* Superior frontal gyrus, orbital*; MFG* Middle frontal gyrus; *ORBmid* Middle frontal gyrus, orbital; *IFGoperc* Inferior frontal gyrus, opercular; *IFGtriang* Inferior frontal gyrus, triangular; *ORBinf* Inferior frontal gyrus, orbital; *ROL* Rolandic operculum; *SMA* Supplementary motor area; *SFGmed* Superior frontal gyrus, medial; *INS* Insula; *HIP* Hippocampus; *AMYG* Amygdala; *CUN* Cuneus; *LING* Lingual gyrus; SOG, MOG, *IOG* Superior, Middle, Inferior occipital gyrus; *FFG* Fusiform gyrus; *PoCG* Postcentral gyrus; *SPG* Superior parietal gyrus; *IPL* Inferior parietal lobe; *SMG* Supramarginal gyrus; *ANG* Angular gyrus; *PCUN* Precuneus; *PCL* Paracentral lobe; CAU Caudate nucleus; *PUT* Putamen; *PAL* Pallidum; *HES* Heschl gyrus; *STG, MTG, ITG* Superior, Middle, Inferior temporal gyrus; *TPOsup* Temporal pole: STG; *TPOmid* Temporal pole: MTG.

**Figure 2:**
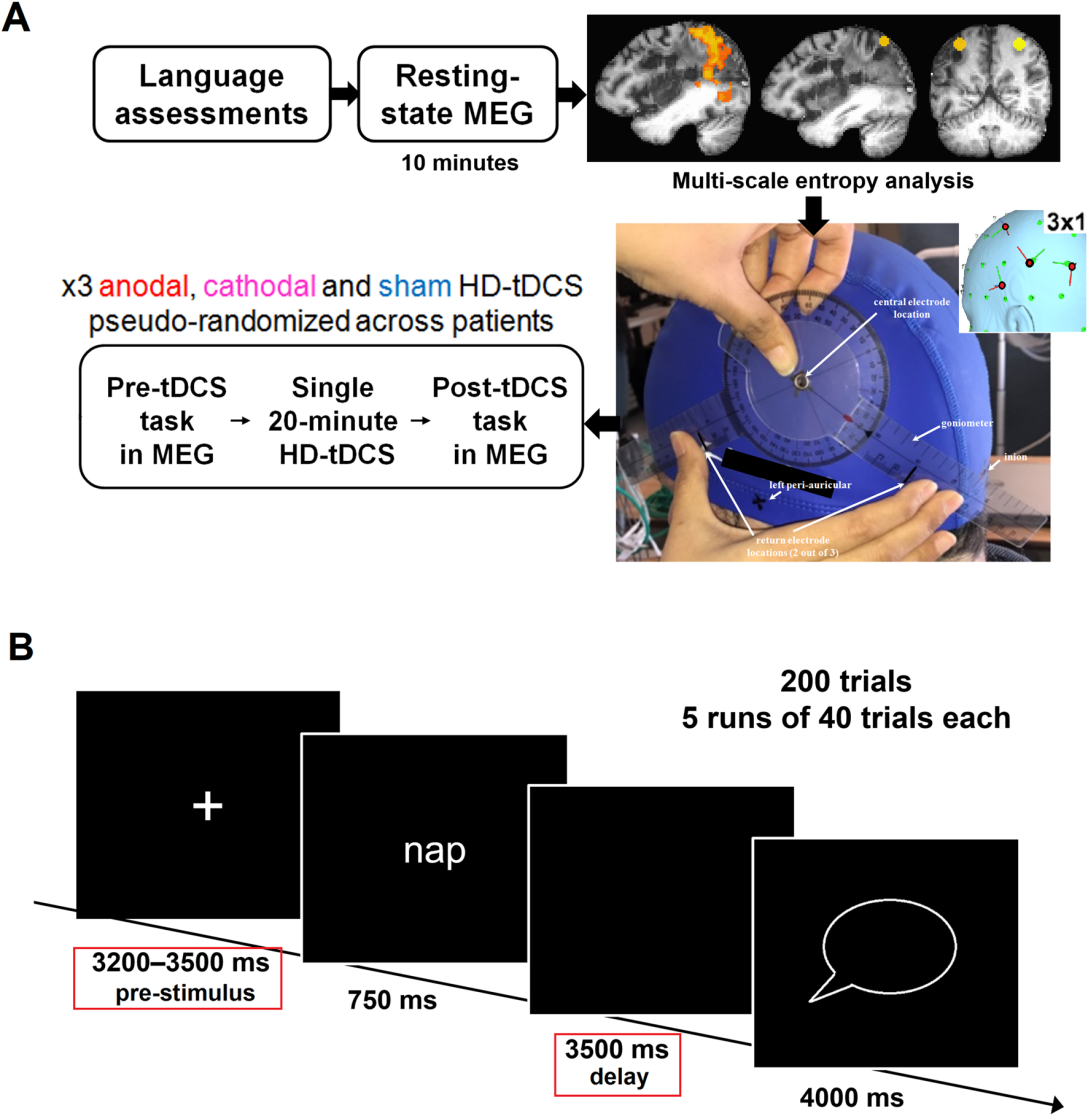
Study design and procedures. (**A**) HD-tDCS was delivered via a 3 × 1 center-surround configuration as shown in the inset (created using simNIBS^69^). A latex swim cap was fitted to the patients, and key locations were marked on the cap directly. The location of the center electrode was individualized based on perilesional abnormalities. During three separate visits, patients completed a delayed word reading task inside the MEG before and after 20 min of stimulation with one of the anode, cathode or sham configurations. (**B**) A sequence of events during a single trial of the delayed word reading task, completed by stroke patients and controls inside the MEG. The MEG analysis for tDCS effects included evaluations of the pre-stimulus (i.e., the fixation) and the delay signals (marked by red boxes) extracted from the stimulation sites.

Figure 1 displays the stroke lesions of individual patients, their sites of stimulation in the left perilesional and right contralesional cortex, and estimated percent damage in atlas-defined regions. Lesions were semi-manually traced, aided by automated segmentation of grey matter, white matter, and CSF, on each patient’s T1-weighted images, and summarized using the automated anatomical labeling (AAL) atlas, consisting of 90 cortical and subcortical regions; cerebellar regions from this atlas were excluded. Percent regional damage as shown in Fig. 1 was computed as number of voxels that were lesioned within an AAL region with respect to the total number of voxels within the same region. Total percent damage as reported in Table 1 was computed as the number of voxels that were damaged with respect to the total number of voxels across all AAL regions. A schematic of the study procedures is shown in Fig. 2A.

**Table 1:** Demographics, clinical variables and sites of stimulation for the stroke patients in this study.

ID	Age (year)	Education (year)	Sex	Time post-onset	Etiology	Aphasia type	% left damage	WAB-AQ	Difficulty of the sentence repetition exercises	Individualized stimulation site		
										Method	Region	Lobe
P1	61	16	M	4 year	Ischemic	Non-fluent	19.8	77.5	Easy	Singleton t-test	PrG/MFG	Frontal
P2	67	16	M	6 year 2 month	Ischemic	Fluent	12.9	75.8	Hard	Singleton t-test	MTG	Temporal
P3	60	12	M	6 year 5 month	Unspecified	Non-fluent	42.1	45.8	Easy	Singleton t-test	SPL	Parietal
P4	62	14	M	5 year	Ischemic	Non-fluent	38.6	52.5	Easy	Singleton t-test	SPL	Parietal
P5	70	20	M	9 year	Unspecified	Anomic	21.3	96.7	Hard	Singleton t-test	IPL/PrG	Parietal
P6	63	14	F	13 year	Hemorrhagic	Conduction	28.7	85.8	Hard	Singleton t-test	MTG	Temporal
P7	71	14	F	9 year 4 month	Hemorrhagic	Anomic	23.4	87.5	Hard	Coarse MSE z-score	MTG	Temporal
P8	34	19	F	4 year	Ischemic	Anomic	19.0	89.2	Hard	Coarse MSE z-score	PoG	Parietal
P9	72	21	M	21 year 4 month	Hemorrhagic	Non-fluent	31.8	34.2	Easy	singleton t-test	STG	Temporal
P10	41	18	M	5 year 9 month	Ischemic	Non-fluent	22.3	59.2	Easy	Coarse MSE z-score	IPL/SMG	Parietal
P11	46	18	F	4 year 2 month	Unspecified	Conduction	20.1	68.3	Hard	singleton t-test	SPL	Parietal
P12	65	12	F	7 year 4 month	Hemorrhagic	Non-fluent		28.3				
P13	68	16	M	4 year 10 month	Hemorrhagic	Anomic	15.5	98.3				
P14	75	18	F	2 year 1 month	Ischemic	Anomic	17.4	100.0				
Mean (SD)	61.1 (12.3)	16.3 (2.8)	8 M	7 year 4 month (4 year 10 month)			24.1 (8.5)	71.3 (23.9)	6/11 hard			6/11 parietal

*WAB-AQ* Western Aphasia Battery-Aphasia Quotient, *M* Male, *F* Female, *SD* Standard deviation, *Unspecified* not clear from medical reports, *PrG* Precentral Gyrus, *MFG* Middle Frontal Gyrus, *MTG* Middle Temporal Gyrus, *SPL* Superior Parietal Lobe, *IPL* Inferior Parietal Lobe, *PoG* Postcentral Gyrus, *ST*G Superior Temporal Gyrus, *SMG* Supramarginal Gyrus.

### Behavioral results

No serious adverse events were reported with HD-tDCS; two patients reported minor itchiness and neck pain/stiffness during anodal- and cathodal-tDCS, and one other patient reported neck pain/stiffness during sham-tDCS.

The mean Western Aphasia Battery-Aphasia Quotient (WAB-AQ; an aphasia severity scale) in the eleven patients was 69.2 (± 19.5; range = 34.2–96.7; Table 1). Six patients received the Hard version of the repetition task, and the rest received the Easy version. The Hard version comprised repeating sentences and the Easy version comprised repeated short phrases (see Supplementary Table 2 for examples). One patient (P09) was excluded from the behavioral analysis because they could not perform the repetition task nor the MEG delayed word reading task; the accuracy at baseline and across all tDCS sessions was 0 for this patient. Mean WAB-AQ significantly differed between patient groups receiving Hard and Easy versions (Wilcoxon Rank Sum test, p = 0.038). AQ was lower in the Easy (53.8 ± 16.1) than the Hard group (81.9 ± 10.9).

Percent damage in the left hemisphere cortex was not significantly different between the patient groups (Hard: 20.9 ± 5.0%; Easy: 30.9 ± 9.8%; p = 0.125; Fig. 1).

#### Baseline vs. post-tDCS repetition performance

**Figure 3:**
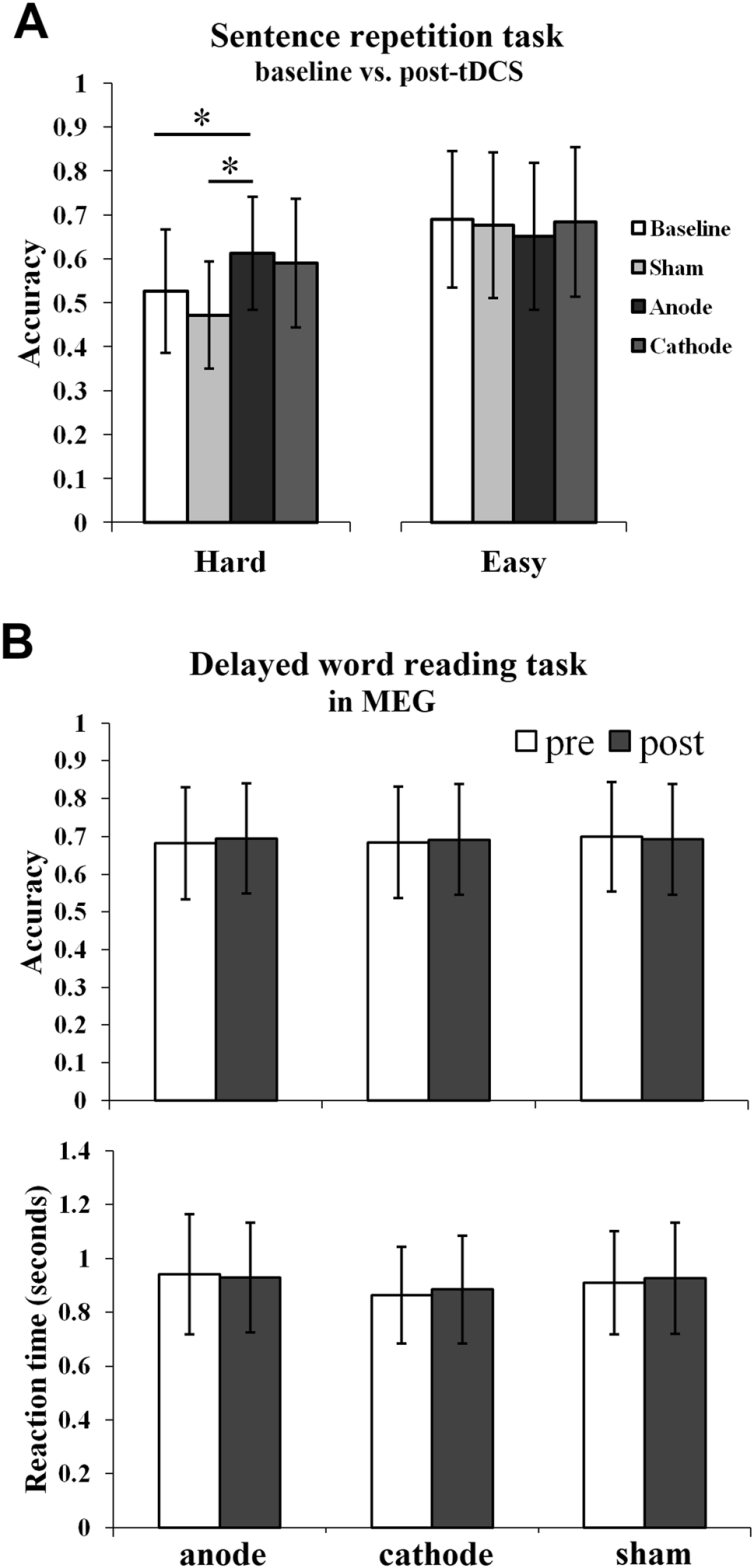
Behavioral changes in stroke patients with tDCS on the repetition and the delayed word reading task. (**A**) Post-stimulation and baseline accuracy on the repetition task in patients receiving the Hard version and in those receiving the Easy version of the task. (**B**) Post- vs. Pre-stimulation delayed word reading accuracy (top) and reaction times in seconds (bottom). Error bars indicate SE and asterisks (*) indicate statistical significance at p < 0.05.

Differences in scoring by the repetition task versions necessitated separate analysis for the effects of tDCS on accuracy by groups. Repetition accuracy in individual patients is provided in Supplementary Table 3. In the Hard group, nonparametric Conover’s tests, corrected for multiple comparisons using the false discovery rate (FDR) method, indicated greater accuracy post-anodal-tDCS (61 ± 25%) compared to both baseline (p = 0.038; 53 ± 23%; Cohen’s *d* = 0.34) and post-sham (p = 0.021; 47 ± 20%; Cohen’s *d* = 0.63) performances (Fig. 3A). Posthoc power calculations revealed that with one-tailed Wilcoxon signed-rank test for matched pairs, 6 participants (in the Hard group) provided 0.36 power, to detect a medium effect size of 0.63.

There was trend toward significant differences after cathodal-tDCS (58 ± 26%) compared to sham-tDCS (p = 0.087) but no differences compared to baseline (p = 0.204). Accuracy differences were not significant in the Easy group (overall accuracy: 67 ± 16%).

#### Delayed word reading task performance

A sequence of events during a single trial of the delayed word reading task, completed by stroke patients and controls inside the MEG is provided in Fig. 2B.

The mean accuracy in ten patients was 69% (± 36) and in controls was 99% (± 0.07). The mean reaction time in patients was 909 (± 636) ms and in controls was 696 (± 196) ms. In patients, the post vs. pre accuracy and reaction times did not differ across stimulation conditions (sham, anode, cathode), as indicated by a non-significant interaction between these variables in a repeated-measures ANOVA (Fig. 3B; see Supplementary Fig. 1 for the effects of word frequency and number of syllables on accuracy in patients).

### MEG results

Based on the individualized site selection methods, six out of the eleven patients were stimulated at a site within the parietal lobe, four within the temporal lobe, and only one within the frontal lobe.

#### Pre-stimulus and delay-period oscillatory and MSE differences between-groups

**Figure 4:**
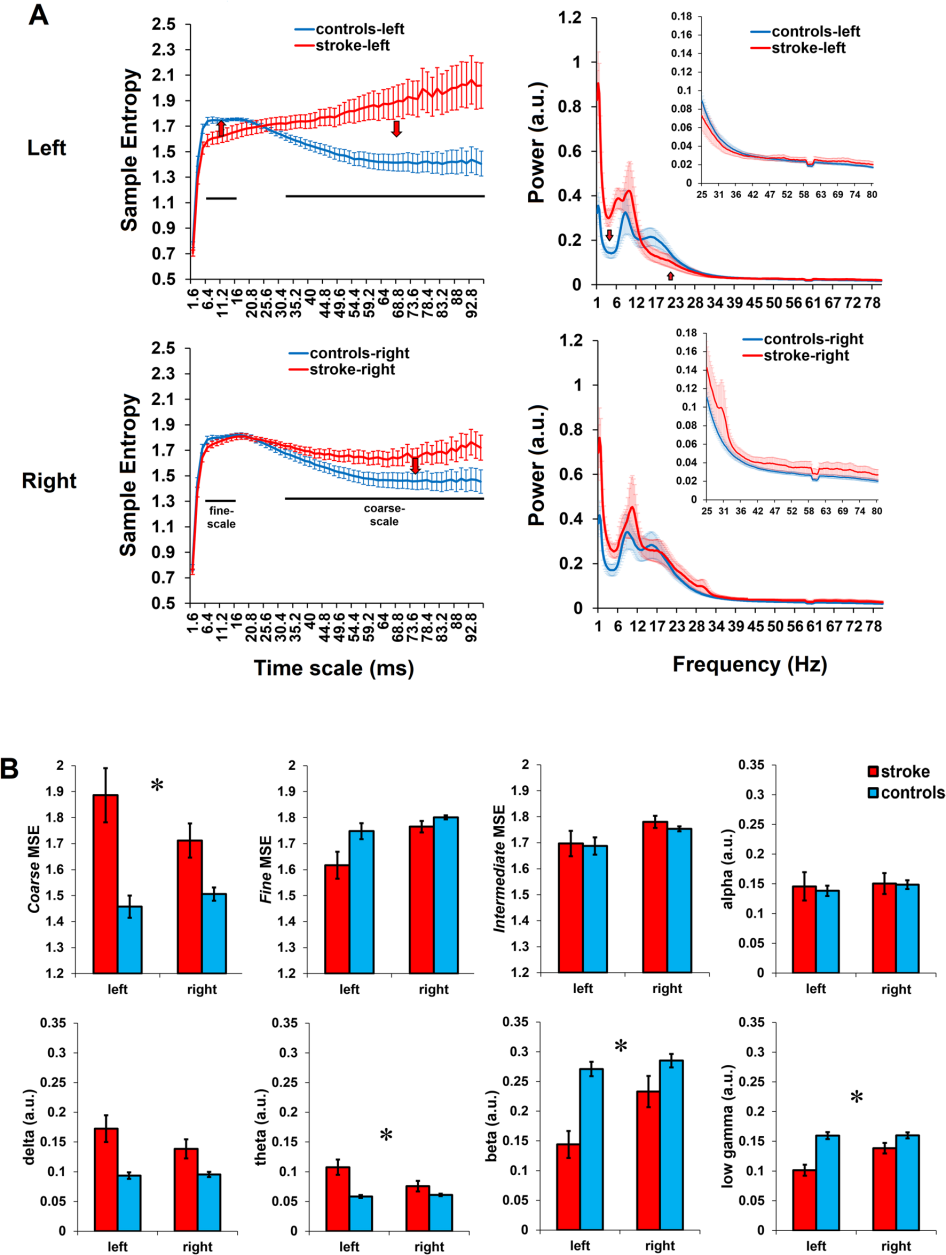
Oscillatory and multi-scale entropy (MSE) differences in the left vs. the right hemispheric stimulation sites extracted from the pre-stimulus and delay period of the delayed word reading task from the pre-tDCS data. The comparisons are between stroke patients and controls. (**A**) Displaying the line plots of sample entropy and power spectral densities to visualize the differences in time scale-dependent signal complexity and spectral properties. With tDCS, we expected normalization of neuronal activity; the hypothesized directions of change are represented by red arrows. (**B**) Differences in mean oscillatory and MSE values between groups and between the stimulation sites. Asterisks (*) indicate significant interaction between group (stroke, controls) and stimulation site (left, right) at p < 0.05 from linear mixed models.

The model with mean fine-scale MSE (marginal/conditional R^2^ = 0.302/0.963) indicated significant fixed effects of group (model estimate b = 17.3; t(20) = 2.1, p = 0.033) and site (b = 22.3; t(20) = 5.2, p < 0.001) but the group × site interaction was not significant (p = 0.10), revealing that fine-scale MSE was lower in patients (1.69 ± 0.15) than controls (1.77 ± 0.07) across both sites, and lower in the left (1.68 ± 0.15) than the right site (1.78 ± 0.06) across both groups (Fig. 4B). Whereas, for coarse-scale MSE (R^2^ = 0.304/0.975), the group × site interaction was significant (b = 16.0; t(20) = 2.3, p = 0.023) indicating higher coarse-scale MSE in the left than the right site in stroke vs. controls (stroke, left: 1.88 ± 0.34; stroke, right: 1.71 ± 0.22; controls, left: 1.46 ± 0.14; controls, right: 1.50 ± 0.08).

The fixed effect of group was significant for delta (b = 28.4; t(20) = 3.4, p = 0.001; R^2^ = 0.284/0.913) but with no difference with respect to the stimulation sites (p = 0.36) or group × site interaction (p = 0.13) was found. Thus, overall delta power was higher in patients (0.15 ± 0.07) than controls (0.09 ± 0.01). The group × site interaction was significant for theta (b = 24.7; t(20) = 3.4, p = 0.001; R^2^ = 0.151/0.953), beta (b = 17.8; t(20) = 3.4, p = 0.001; R^2^ = 0.459/0.970) and low-gamma (b = 24; t(20) = 3.3, p = 0.001; R^2^ = 0.483/0.949) power values. Together these results indicated stroke patients were specifically different from controls in the left hemisphere vs. the right hemisphere in the form of higher theta (stroke, left: 0.10 ± 0.04; stroke, right: 0.07 ± 0.03; controls, left: 0.06 ± 0.01; controls, right: 0.06 ± 0.01), lower beta (stroke, left: 0.14 ± 0.07; stroke, right: 0.23 ± 0.09; controls, left: 0.27 ± 0.04; controls, right: 0.28 ± 0.04) and lower low-gamma (stroke, left: 0.10 ± 0.03; stroke, right: 0.14 ± 0.03; controls, left: 0.16 ± 0.02; controls, right: 0.16 ± 0.02) (Fig. 4). No differences with respect to group and/or site were found in the alpha-band or the intermediate MSE scale (see Supplemental Material, and Supplementary Fig. 2).

The model details and code snippet from R are provided in Supplementary Table 4.

#### Relationship between pre-stimulus oscillatory dynamics and language dysfunction in stroke

Next, we interrogated whether the MSE and relative power in left/perilesional sites of stimulation were associated with the degree of language impairment. If related, we can draw associations between perilesional rsMEG abnormalities, defined in a data-driven manner (i.e., peak or center of mass of abnormalities), and a behavioral measure of aphasia severity (Supplementary Fig. 3A). We found significant positive relationships between beta (rho = 0.74, p = 0.012) and low-gamma power with WAB-AQ (rho = 0.69; p = 0.023), and a weak relationship between fine-scale MSE and WAB-AQ (rho = 0.59; p = 0.061), indicating that more beta and low-gamma power and possibly fine-scale MSE are related to better language function (i.e. or higher AQ scores, less aphasia severity). For coarse MSE (rho = − 0.65, p = 0.034) and lower frequency ranges (delta: rho = − 0.72, p = 0.015; theta: rho = − 0.46; p = 0.150), a negative relationship was found with WAB-AQ, indicating that higher delta power relates to higher aphasia severity (i.e. lower WAB-AQ scores). Relationships with alpha power (rho = 0.44, p = 0.182) and intermediate-scale MSE (rho = 0.34, p = 0.313) were not significant. These findings suggest that perilesional slowing (high low-frequency activity and low high-frequency activity) and time scale-dependent changes in MSE (high coarse-scale MSE and weakly low fine-scale MSE) in individually selected left stimulation sites are associated with language dysfunction. Thus, we expect that the changes in response to tDCS would be in the direction indicating better language outcome, i.e., reduced low-frequency activity, increased high-frequency activity, reduced coarse-scale MSE, and increased fine-scale MSE in perilesional areas.

Next, informed by between-group differences in the right hemisphere stimulation sites (homologous to the perilesional sites), we examined whether power and MSE from these sites were associated with language dysfunction severity (Supplementary Fig. 1B). We found a negative relationship of WAB AQ with coarse-scale MSE (rho = − 0.69, p = 0.023) and weakly with theta (rho = − 0.59, p = 0.061), along with a positive relationship with beta (rho = 0.82, p = 0.004). Correlations with delta (rho = − 0.46, p = 0.154), and fine-scale MSE (rho = 0.26, p = 0.435) and low-gamma (rho = 0.10, p = 0.775) were not significant.

#### Effects of tDCS on spectral and MSE measures in stroke

The average transfer time to MEG across all stimulation sessions and patients after the end of tDCS was 15.7 (± 3.2) minutes.

**Figure 5:**
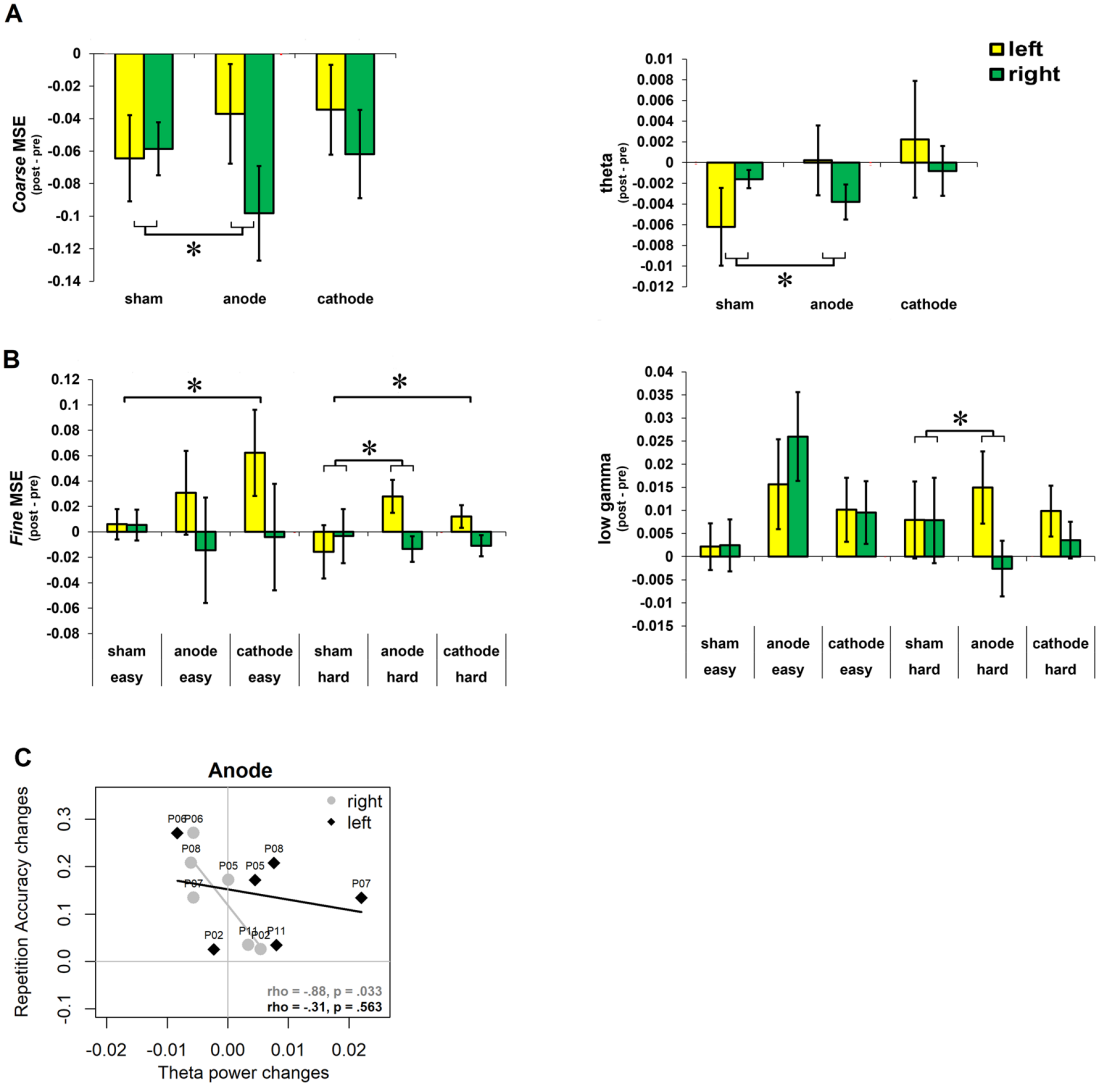
Effects of tDCS on spectral and MSE measures in stroke patients. Post vs. pre-stimulation differences are presented on the y-axis, separated by stimulation conditions (**A**) and Hard vs. Easy versions of the repetition task (**B**) on the x-axis. (**A**) A significant two-way interaction between stimulation sites (left, right) and stimulation conditions (sham vs. anodal-tDCS) was found for coarse-scale MSE and theta. Larger reductions in these measures were found in the right site after left anodal-tDCS. (**B**) Significant three-way interaction was found for anodal-tDCS among the stimulation sites (left, right), conditions (sham vs. *anodal-tDCS*) and training task version (Hard, Easy) for fine-scale MSE and low-gamma. Increases in these meaures were found in the left stimulation site in the Hard group after anodal tDCS. Two-way interaction between sites (left, right) and stimulation conditions (cathodal-tDCS vs. sham) was also found, which revealed increases in fine-scale MSE in the left site after cathodal-tDCS compared to sham-tDCS across both Easy and Hard groups. Error bars indicate SE. Asterisks (*) indicate significance at p < 0.05 from linear mixed models; refer to the text for more details. (**C**) Scatter plot indicating a relationship between changes in repetition accuracy and theta power changes after anodal-tDCS with respect to sham-tDCS for left and right stimulation sites.

The model outputs revealed a significant anodal-tDCS × site interaction for post minus pre differences in theta (b = 40.0; t(90) = 3.0, p = 0.003; R^2^ = 0.156/0.374) and coarse-scale MSE (b = 33.8; t(90) = 2.6, p = 0.010; R^2^ = 0.113/0.423), indicating reductions after anodal-tDCS, particularly in the right site, compared to sham-tDCS (Fig. 5A).

The model output for fine-scale MSE (Fig. 5B) indicated a three-way interaction among anodal-tDCS × site × training-task version (fine-scale MSE: b = 54; t(81) = 2.53, p = 0.011; R^2^ = 0.153/0.694), revealing selective increase in fine-scale MSE after anodal-tDCS paired with the Hard task in the left site compared to sham-tDCS paired with the Hard task. The model also indicated a significant two-way interaction between cathodal-tDCS × site (b = 24.9; t(81) = 2.33, p = 0.020), revealing increases in fine-scale MSE in the left site after cathodal-tDCS, irrespective of the training task version, as compared to sham-tDCS.

The model output for low-gamma power (Fig. 5B) also indicated a three-way interaction among anodal-tDCS × site × training-task version (b = 51.8; t(99) = 2.4, p = 0.015; R^2^ = 0.125/0.621), revealing selective increase in low-gamma power after anodal-tDCS paired with the Hard task in the left site compared to sham-tDCS with the Hard task. Unlike fine-scale MSE, low-gamma power was not affected after cathodal-tDCS. The differences in delta and beta power values were not significantly affected.

Model details and R code snippet are provided in Supplementary Table 5, and mean changes with tDCS in individual patients are provided in Supplementary Table 6.

#### Relationship between the effects of tDCS on MEG measures and changes in behavioral performance

To evaluate whether tDCS and behavioral changes were related, we ran correlation analyses between improvements in repetition accuracy, i.e., on the task conducted before and after tDCS and changes in MEG parameters after anodal-tDCS (compared to sham) and cathodal-tDCS (compared to sham). These analyses were conducted in the Hard group only for a practical reason: The stimuli used (phrase vs. senstences) in the Hard vs. Easy versions necessitated different scoring approaches rendering non-comparable scores between versions. MEG parameters for correlations included theta power, coarse MSE, low-gamma power and fine MSE compared to sham, whereby data from left and right stimulation sites were evaluated separately.

Significant correlations were found between changes in repetition accuracy and theta power changes in the right (rho = − 0.88, p = 0.033) but not left (rho = − 0.31 p = 0.563) stimulation site after anodal-tDCS (Fig. 5C). No other correlations were significant (all p > 0.05).

## Discussion

Resting-state MEG signal complexity analysis allowed individualized selection of targets for high-definition tDCS in eleven chronic post-stroke patients with aphasia. Perilesional areas exhibiting abnormal rsMEG complexity were targeted with anodal-tDCS and the homolog (right-hemispheric) areas were targeted with cathodal-tDCS during separate 20-min sessions, which were both compared with sham-tDCS. Anodal-tDCS resulted in significant improvements in repetition accuracy but these improvements were dependent on the training task difficulty level, whereas homolog cathodal-tDCS was only weakly effective. Thus, rsMEG guided selection of perilesional targets with a focal form of anodal-tDCS can be an efficacious approach for treatments in chronic aphasia. There are caveats related to the training task (Hard, Easy) and/or language impairment severity that we discuss in detail in the sections below.

The neuromodulatory changes evaluated with MEG after both active-tDCS conditions (compared to sham-tDCS and pre-stimulation conditions) pointed to reduction of oscillatory abnormalities based on the assessment of local changes directly under the stimulation electrode, as revealed by signal complexity/MSE and spectral power analyses. Anodal-tDCS targeted to perilesional areas increased low gamma and signal complexity in the fine-scale MSE range and reduced contralateral theta and signal complexity in the coarse-scale MSE range. Out of these changes, changes to theta power in contralateral areas after perilesional anodal-tDCS were associated with improved repetition accuracy as compared to sham-tDCS in the Hard group. This indicated that more theta reductions in right areas, contralateral to the left stimulation site, were related to greater accuracy changes. After cathodal-tDCS, increases in fine-scale MSE were found across the Hard and Easy groups but these changes were not found to be correlated with repetition accuracy changes compared to sham.

Our study is one of the few studies using HD-tDCS in post-stroke aphasia, and the first one that we know of examining the ensuing neurophysiological changes with MEG. Richardson and colleagues (2015) compared conventional tDCS and 2 × 2 anode and cathode HD-tDCS montage on the left temporal areas and found equivalent benefits in picture-naming performance with both montages^44^. A recent study by Fiori and colleagues (2019) tested the efficacy of 1 mA vs. 2 mA cathodal-tDCS on the right Broca’s area in a 4 × 1 configuration and found significant improvements in verb naming but only with 2 mA cathodal-tDCS^45^. Building on these studies, we provide a direct comparison of behavioral and physiological effects between anodal-tDCS and cathodal-tDCS within the same patients using a focal, 3 × 1 tDCS configuration.

A cued repetition task was administered during stimulation, the difficulty of which was adjusted to sufficiently engage patients with different types of language impairments. The differences in task difficulty and/or aphasia severity may have affected the response to tDCS. An increase in repetition accuracy was found in the Hard group after anodal-tDCS, but not in the Easy group; relative to the baseline performance, percentage increase in accuracy was 15% and 11% after anodal-tDCS and cathodal-tDCS, respectively, in the Hard group. Patients in the Easy group (WAB-AQ: 53.8 ± 16.1) were more impaired than those in the Hard group (81.9 ± 10.9). One possibility is that the Easy training task involving repetition of phrases was not sufficiently intensive, leading to minimal boost in performance with tDCS in the Easy group. Prior studies have indicated that performance changes with tDCS can be maximized by pairing it with tasks that not only recruit the brain areas targeted with tDCS but also with tasks that are cognitively demanding and challenging^46^. Second possibility is that a single 20-min session in more impaired patients could not effectively induce performance changes. Thus, it remains to be shown whether these patients would respond to more intensive language therapy paired with protracted tDCS regimens.

We replicated our previously reported findings^23,24^ related to increased coarse-scale and reduced fine-scale entropy and spectral slowing in perilesional areas. In the current study we also conducted a more detailed comparison of power spectra and MSE curves between stroke patients and matched controls (Fig. 3), whereas these comparisons in our previous studies focused on perilesional vs. contralateral activity within stroke patients. The physiological bases of perilesional slowing that these measures indicate remain uncertain, but candidate mechanisms include chronic hypoperfusion, white matter disconnection, and local stroke-related physiological disruption that may have selectively damaged neural architecture without a structural lesion^24,25^. Whatever the mechanism, we hypothesized that perilesional abnormalities are in principle partially reversible given intervention. Extending these findings, we found patterns of abnormal activity in the intact, contralateral right hemispheric areas although to a lesser degree, as indicated by significant interactions between hemisphere and power/MSE values in our between-group analyses (Fig. 3B). We also demonstrated that these perilesional and contralateral abnormalities were related to language impairment severity, indicating a link between pathological oscillatory activity and language impairments (Fig. 4). Because we did not include neuropsychological assessments of other, non-language domains, or motor assessments, it is difficult to comment on the specificity of oscillatory abnormalities to language dysfunction in our patients with aphasia. This remains to be explored in future studies.

Nonetheless, current findings aided us in defining what normalization with tDCS might look like; changes in response to tDCS would be in the direction indicating better language outcome as determined from our correlational analyses, before any stimulation. As such normalization would indicate reduced coarse-scale and increased fine-scale complexity, and spectral speeding as indicated by increased high-frequency and reduced low-frequency activity. After tDCS, the local rsMEG changes, i.e. changes occurring at the stimulation site and in the contralateral site, pointed toward normalization with converging evidence from MSE and spectral power estimates. Anodal-tDCS acted by increasing the fine-scale MSE and high-frequency low-gamma activity at the left stimulation site in the Hard group, who *did* benefit behaviorally from stimulation. Anodal-tDCS also decreased the coarse-scale MSE and low-frequency theta activity in the contralateral site independent of the training task difficulty, i.e. changes were comparable across the Hard and Easy groups. Cathodal-tDCS increased fine-scale MSE in the left site, however unlike anodal-tDCS, these changes were independent of the training task difficulty. Interestingly, the induced anodal-tDCS changes in theta manifested in the right site and induced cathodal-tDCS changes manifested in the left site, the sites contralateral to the respective hemispheric sides of stimulation. Together these results suggest reversal of perilesional abnormalities after both perilesional anodal-tDCS and contralateral cathodal-tDCS, as well as normalization in the contralateral areas but only after anodal-tDCS.

Importantly, this latter change—theta power reductions in the right site after left anodal-tDCS, was the only measure that was significantly correlated with increased repetition accuracy in the Hard group. Ongoing work in signal analysis is characterizing the relationship between MSE and spectral power measures together with brain network dynamics, which may yet produce new algorithms to optimize individualized targeting and detection of induced changes with tDCS^30,47,48^. As far as current study findings are concerned, we know that MSE quantifies predictability of a signal such that higher entropy indicates lesser predictability or more signal complexity. Conceptually, however scale-dependent MSE and spectral measures are hypothesized to reflect network dynamics of information processing^30,47^. The signal complexity at finer-scales and spectrally higher-frequencies are thought to represent functional segregation, and coarser-scales and low-frequencies to represent functional integration^49–51^. One interpretation of our between-group results is that there is reduced functional specialization and increased integration after stroke compared to matched controls. The tDCS induced changes could then be interpreted as increased specialized processing within the perilesional areas accompanied by reduced distributed processing across the right areas, particularly after anodal-tDCS. The spectral results mirrored MSE results and could be interpreted in the same way. The reduced distributed processing involving right areas after anodal-tDCS may be linked to observed improvements in repetition accuracy in the Hard group (Supplementary Fig. 3). Future studies using direct measures will further characterize the link between MSE/spectral changes and network configuration and communication properties with tDCS and those associated with successful treatment outcomes.

Given that the size and location of stroke lesions vary considerably across patients, the boundary of lesions, which delineate perilesional areas and homologous areas in the contralesional hemisphere will also vary considerably across patients. Thus, placement of tDCS electrodes on a predefined region, particularly with more focal forms of tDCS, may not consistently target and may in some cases miss the perilesional areas across patients. Functional ‘localizers’ of perilesional activity using linguistic task-based fMRI have been used successfully, but this may not work when perilesional areas fail to exhibit task-related activity due to their functionally compromised status. In the current study, we took a different approach using MSE derived from rsMEG, which sensitively marks perilesional areas, allowing these areas and their homologous areas in the right hemisphere to be targeted with tDCS on an individual basis. Our approach using resting-state/task-free analyses for locating tDCS targets can better generalize across aphasia survivors, presenting with heterogeneous language impairment profiles and severity, than approaches that depend on performance of specific linguistic tasks, particularly in patients unable to complete linguistic tasks. It also circumvents the need to control for task performance and strategy differences across participants.

Our approach does have some limitations. We focused primarily on MSE differences to determine the location of peak perilesional abnormalities compared to healthy controls. In 3 out of the 11 patients (Table 1), statistical comparisons of MSE with healthy controls were not significant, and so we used the within-participant z-score approach instead to localize perilesional abnormalities in these patients. It is unclear from their clinical profiles why the first approach was not successful. To ensure more consistency in future applications, we recommend that stimulation sites should be determined based on converging results from spectral power and MSE differences with respect to healthy controls. In cases where statistical comparisons are not successful, converging findings from within-participant z-score approach can be employed. Lastly, clinical factors such as language impairment types, for example, broad classification into fluent or nonfluent and/or specific impairments in semantic or phonological processing, related to retrieval or selection, together with known functional neuroanatomy and MEG abnormalities can all be taken into account for the selection of stimulation sites. Another limitation of our study is the small sample sizes, particularly in assessment of behavioral effects of tDCS because of the grouping by repetition task difficulty. Future larger scale studies will need to be conducted to confirm our proof-of-concept study findings.

Our MEG findings add to the growing body of evidence suggesting normalization of spontaneous and task-induced fluctuations with both left anodal- and right cathodal-tDCS. Future applications of MEG and tDCS should elucidate the link between these immediate physiological changes and long-term behavioral benefits after protracted tDCS treatments. Such links may reflect “consolidation” of the neural changes, or can reflect homeostatic metaplasticity instead whereby the brain may resist the induced changes by changing in the opposite direction.

## Methods

### Participants

Fourteen patients with aphasia participated in the study. Two patients (P13 and P14) were excluded because of ceiling performance determined based on aphasia severity scale—the Western Aphasia Battery (Table 1). One patient opted out after initial visits (P12; Table 1). Eleven patients completed all the study requirements (mean ± standard deviation: age: 58.8 ± 12.8 years; education: 16.5 ± 2.6 years; 7 males; Table 1). The sample size was estimated using a priori power calculations on repeated measures ANOVA (within factors) consisting of 2 groups and 3 measurements per group, which revealed that total of 10 participants would provide 0.80 power to detect small-to-medium effect size of 0.55 at alpha = 0.05.

Patients suffered a single left-hemispheric stroke at least 4 years prior to the study (stroke onset: 8.0 ± 5.2, 4–21 years). Five patients suffered an ischemic stroke, 3 a hemorrhagic stroke, and 3 had an unspecified etiology. The diagnosis of aphasia was made by a speech-language pathologist and/or a board-certified neurologist, and further verified based on clinical presentation, narrative speech samples and standardized linguistic tests.

Ten age- and sex-matched healthy controls were also recruited (age: 60.4 ± 14.4 years; education: 17.3 ± 1.6 years; 6 males; Supplementary Table 1). Controls completed the delayed word reading task inside the MEG but did not participate in the tDCS arm of the study. All controls had T1-weighted structural MRIs on file from previous studies.

Participants were right-handed (pre-stroke), native speakers of English, and had normal hearing and normal or corrected-to-normal vision. All stroke patients retained sufficient language comprehension capacity to consent and follow task instructions. Exclusion criteria were other neurological diseases, language disorders (for controls), head traumas or brain surgery, epilepsy, severe psychiatric disorders, unstable or poor health, and any contraindications for tDCS and/or MEG^52^.

This study was approved by the Research Ethics Board at Baycrest Health Sciences. All participants gave their written informed consent according to the Declaration of Helsinki prior to the study and were compensated for their participation.

### Study procedures

Figure 2A provides a schematic of the study procedures. All participants completed a series of language assessments during their first visit. Aphasia severity and type were determined based on the Western Aphasia Battery (WAB). Patients with very mild aphasia (WAB-aphasia quotient > 98) were excluded from further participation. The difficulty version of the repetition training task (during stimulation) and repetition assessment task (performed before/after stimulation) was determined based on individual patients’ aphasia type. ‘Easy’ version, which included repetition of phrases, was administered in patients with non-fluent aphasia, and the ‘Hard’ version with repetition of full sentences was administered in all other aphasia types (Conduction, Anomic, Fluent). Next, patients completed the baseline repetition task with either phrases (Easy) or sentences (Hard), as determined earlier. This baseline assessment included all the stimuli that we planned to test the patients on immediately after stimulation of each type. The stimulus sets were devised such that stimuli used for training did not overlap with stimuli used during post-stimulation assessments. But the assessment stimuli were tested twice, once during baseline where all stimuli were tested, and once with a subset of stimuli post-stimulation of each kind.

Next, during one of the baseline visits, patients completed 10 min of rsMEG with eyes open. MRI scans were also acquired during this visit. RsMEG data were analyzed for relative power and MSE measures, which informed the locations of stimulation in each individual patient. Once stimulation sites were mapped, patients visited the lab on three separate days for tDCS-MEG sessions. During the first visit, a latex swim cap was fitted and stimulation sites were marked directly on the cap, guided by a neuronavigation software loaded with patients’ own structural MRI scans.

During each stimulation visit, patients performed a delayed word reading task (Fig. 2B) before and after stimulation. Patients received stimulation with one of the following three types: left anodal-tDCS, right cathodal-tDCS or sham-tDCS. The order of stimulation type was randomized across patients, and the time interval between stimulation visits was set to be at least 48 h. During stimulation, patients completed a cued version of the repetition task, whereby the stimuli were either phrases or sentences, as determined earlier. Immediately after the stimulation ended, they completed an uncued repetition task, using a set different from the one they were trained on during stimulation; a different set was used after each stimulation type. After this assessment, the patient was transferred to the MEG room, where they completed the post-stimulation delayed word reading task. After post-tDCS MEG ended, patients completed a tDCS-related adverse event questionnaire.

Age-, sex- and education-matched healthy controls were recruited after the recruitment of stroke survivors ended. They came into the lab once and completed the delayed word reading task with MEG, without any stimulation.

### Language assessments

Stroke patients completed a neurolinguistic battery (Table 2), consisting of the Western Aphasia Battery (WAB; Revised Bedside form)^53^ for classification of the aphasia-type and determining severity, the 60-item Boston Naming Test (BNT)^54^ for confrontational naming assessment, the Peabody Picture Vocabulary Test (PPVT)^55^ for receptive lexical semantics and vocabulary knowledge, and the non-word reading test from the Psycholinguistic Assessments of Language Processing in Aphasia (PALPA)^56^ for assessment of reading and phonological processing.

**Table 2:** Language assessment scores.

	BNT		PPVT	PALPA8	Western Aphasia Battery (Bedside)						
	Raw	Scaled			Content	Fluency	Comprehension	Commands	Repetition	Naming	Aphasia Quotient
P1	30	1	92	3.3	8	4	10	7	7.5	10	77.5
P2	39	1	85	93.3	10	8	10	4	5	8.5	75.8
P3	13	1	77	NT	5	2	9	2	5	4.5	45.8
P4	9	1	96	NT	6	2	10	2	6	5.5	52.5
P5	50	6	114	93.3	10	9	10	10	9	10	96.7
P6	35	2	97	40.0	10	8	10	8	7	8.5	85.8
P7	53	7	103	50.0	9	8	10	8	8	9.5	87.5
P8	60	13	104	70.0	10	7	9	9	8.5	10	89.2
P9	0	1	95	NT	3	1	8	6	1.5	1	34.2
P10	18	1	73	NT	6	2	8	7	5	7.5	59.2
P11	39	2	84	33.3	8	15	9	5	5	9	68.3
P12	5	1	70	NT	1	3	7	1	3	2	28.3
P13	46	4	100	86.7	10	10	9	10	10	10	98.3
P14	49	6	96	90.0	10	10	10	10	10	10	100.0
Mean (SD)		3.4 (3.54)	91.9 (12.59)	40.0 (40.0)	7.6 (2.95)	5.6 (3.27)	9.2 (0.97)	6.4 (3.13)	6.5 (2.55)	7.6 (3.10)	71.4 (23.91)

BNT = Boston Naming Test^54^. Scores scaled based on age and years of education; PPVT = Peabody Picture Vocabulary Test^55^; PALPA = Psycholinguistic Assessments of Language Processing in Aphasia (Kay et al., 1992), PALPA 8 = total score on nonword reading subtest; Western Aphasia Battery- Bedside version^53^, Content = Spontaneous Speech Content, Fluency = Spontaneous Speech Fluency, Comprehension = Auditory Verbal Comprehension, Commands = Sequential Commands, NT = Not tested,Bedside Aphasia Quotient (WAB-AQ) was determined by summing the Speech Content, Fluency, Auditory Verbal Comprehension, Sequential Commands, Repetition, and Object Naming scores, dividing the sum by 6 and then multiplying the result by 10.

### Sentence repetition exercises

Two versions of the training task were designed to ensure that it was sufficiently engaging across patients with varying types of language impairments. Non-fluent patients received an easier (“Easy”) version of the task and other patients received a relatively more difficult version (“Hard”). Supplementary Table 2 provides an example set of the stimuli. Each set in the Easy version consisted of six stimuli/segments. Patients were expected to repeat single words or word phrases, which were linked together to form a full sentence in later segments. Each set in the Hard version consisted of five or six full sentences that were grouped together to form a cohesive story or a conversation piece. Patients were expected to repeat a full sentence during each segment. A four-step procedure, described in Marangolo et al.^22^, was used *during tDCS* to progressively help the patient to repeat the sentences or phrases correctly^22^, see Supplementary Material for more details. The experimenters (both those who administered and those who scored the task) and the patient were blinded to the tDCS polarity.

For each version, nine sets were designed for training. Another nine sets, non-overlapping with the sets used for training, were designed for baseline and post-tDCS assessments. All nine of these latter sets were administered during baseline, and different three sets out of these nine were then administered post-tDCS of each type. The order of the post-tDCS sets was pseudo-randomized across patients.

Accuracy was computed as a proportion of the number of verbatim recalls by the total number of words in the original sentence/phrase. For the Hard version, extra points were given based on the number of gist words/phrases. See Supplementary Information for more details on scoring.

### High-definition transcranial direct current stimulation

HD-tDCS was delivered for 20-min using a 3 × 1 center-surround configuration, comprising of one center electrode surrounded by three return electrodes^57^ with anode or cathode polarity. HD-tDCS was administered with a multichannel transcranial electrical stimulator (neuroConn DC-STIMULATOR MC, Ilmenau, Germany) according to established guidelines^7^ and procedures^58^. The current intensity at the center electrode was 2 mA for active stimulation. For sham stimulation, the center electrode was the anode, and current intensity was ramped up to 2 mA and back down to 0 mA within the first 30 s; the placement of electrodes for sham was on the right side for 6 and left side for 5 participants.

The location of the center electrode was determined individually based on patients’ rsMEG abnormalities in the perilesional areas (described in more detail below). Using AFNI (https://afni.nimh.nih.gov/; Cox^59^), a 3D sphere with a radius of 8 voxels was placed on the region demonstrating abnormalities, either at the peak or at the center of mass, depending on the location that was more convenient for surface-based targeting. Another sphere was placed in the contralateral homolog region as described in the sections below. These spheres and the T1-weighted MRI scan of the patient were uploaded into the Brainsight Neuronavigation system (Rogue Research, Montreal), and were used to identify the stimulation sites on the scalp. The location of the center electrode, matching the scalp stimulation site, was marked on a regular latex swim cap (Adult Platinum Ultra, Leader Sports, Hilsinger Co Hilco Canada, St Laurent, QC, Canada) fitted to the patient. The locations of the three return electrodes were marked on the cap at a radius of 7 cm and at 120° angle from one another; the surrounding electrodes were placed so that 2 out of the 3 were anterior and 1 electrode was posterior to the center electrode. Four HD-tDCS plastic electrode holders (model HD1, Soterix Medical Inc., New York, NY) were then attached to the cap at these locations, fitted into small holes in the cap and held in place by the latex cap’s elasticity. We used Signa gel and sintered Ag/AgCl chloride ring electrodes (Stens Corporation, San Rafael, CA; internal diameter = 0.5 cm, external diameter = 1.2 cm), as prescribed in Minhas et al.^60^.

In order to ensure that the latex cap was fitted the same way across all stimulation visits, landmarks such as the left and right peri-auricular points, inion and vertex were marked on the cap during the first stimulation visit. The distance between the edge of the cap resting on the patient’s forehead was also noted. During each subsequent stimulation visit, the cap was fitted and aligned to these points before prepping the skin and the electrodes.

### MRI acquisition

MRI was carried out on a 3-T scanner (Siemens TIM Trio). For MEG source localization, we acquired a T1-weighted MPRAGE image (1 mm isotropic voxels, TR = 2000 ms (ms), TE = 2.63 ms, FOV = 256 × 256 mm^2^, 160 axial slices, scan time 6 m, 26 s). MR-visible markers were placed at the fiducial points for accurate co-registration with MEG, aided by digital photographs.

### MEG acquisition, head modeling and source analysis

MEG signals were recorded with a 151-channel whole-head system with axial gradiometers (VSMMedTech, Coquitlam, Canada) sampled at 1250 Hz, with online synthetic 3rd-order gradient noise reduction. Head position inside the MEG helmet was monitored using three fiducial coils placed at the nasion, left and right pre-auricular points. The head positions measured before and after the 10-min resting-state run and after each run of the MEG task (about 12-min long) were averaged. The T1 image was spatially transformed into the MEG coordinate space and skull-stripped. A 3D convex hull approximating the inner skull surface was constructed, and multi-sphere head models tangential to the hull surface^61^ were computed for both the resting-state and task-MEG data.

MEG source analysis was conducted using a scalar beamformer, Synthetic Aperture Magnetometry (SAM) implemented in CTF software (Port Coquitlam, British Columbia, Canada) supplemented with in-house MATLAB scripts. SAM is a scalar beamformer, in which a nonlinear optimization technique is used to select one direction of current flow at each voxel to maximize dipole power. SAM provides a series of sensor weights computed so as to pass signal from a dipole located in the target voxel, while minimizing signal power from all other locations. SAM weights were computed for each whole-brain grid location spaced 10 mm apart for resting-state and 7 mm for the task data. For resting-state, the weights were multiplied with the original sensor time series data to yield a spatially filtered time series signal at each voxel (10 mm^3^). Normalized weights were used to render virtual signals in dimensionless units of signal-to-noise ratio, with noise power estimated as the lowest singular value of the sensor covariance matrix. For task, normalized power differences (SAM pseudo-T)^62^ were computed at each voxel (7 mm^3^). MEG source-level estimates were normalized into MNI space using a nonlinear warp in ANTS software^63^.

### Resting-state MEG processing and analysis

Raw rsMEG recordings of 10-min were screened for artifacts, rejecting segments containing obvious signal disruptions (< 1% of all data). Signals were down-sampled to 625 Hz, divided into 2.5-s epochs, band-pass filtered from 0–100 Hz and then subjected to SAM source analysis. Power spectral densities of the voxelwise virtual signals were computed using the multitaper method. For multi-taper spectral analysis, the time half bandwidth (NW) parameter was set to 3, which resulted in 5 (2*(NW)-1) discrete prolate spheroidal or Slepian sequences for multitaper computations. Whole-brain relative power estimates were computed for delta (1–4 Hz), theta (4–7 Hz), alpha (8–12 Hz), beta (15–30 Hz) and low-gamma (25–50 Hz) frequency bands, as a ratio of the total power from 0.3–100 Hz.

Following the methods in our previously published studies, MSE^23,24^ was computed for each voxel’s virtual signal with the pattern length parameter *m* = 2 and the tolerance parameter *r* = 0.2 from 1–60 scales, corresponding to time scales of 1.6 to 96 ms. The sample entropy values were averaged between 4–10 (6.4 to 16.0 ms; referred to as fine), 11–20 (17.6 to 32.0 ms; intermediate) and 21–60 (33.6 to 96 ms; coarse) scales to generate whole-brain MSE maps. Based on the relationship between frequency and MSE time scales described in Courtiol et al.^30^, Fs/(2 × τ_sf_), where Fs = sampling frequency and τ_sf_ = time scale, fine-scale MSE corresponds to frequencies between 31–78 Hz, intermediate-scale corresponds to 16–28 Hz, and coarse-scale corresponds to 5–15 Hz. Normalized, z-score MSE maps were computed per participant by comparing voxelwise values against the mean and SD across all the voxels.

Both relative power and MSE maps were warped into MNI space and interpolated to achieve 5 mm^3^ resolution for statistical comparisons.

### Individualized stimulation site selection

Two single-subject mapping approaches were employed to identify dysfunctional perilesional areas and to localize the stimulation sites^23^: singleton independent t-tests, and within-subject z-score analysis. Singleton analysis compared an individual patient’s MSE maps at each time-scale with corresponding maps of a group of 24 older controls (age: 67.3 ± 9.8 years); this dataset in older controls was collected as part of a different study, results of which are published elsewhere^64^. The peak or the center-of-mass of the top-ranked clusters that fell within the perilesional cortex and the ones that were consistently different between-groups across 5 or more time scales (false discovery rate corrected, q < 0.05) were selected (Table 1). Where singleton t-test comparisons were not consistently significant (in 3 out of 11 patients), within-subject, z-score coarse-scale MSE maps were used for detecting abnormalities by self-comparisons. These maps were thresholded at 30% of the absolute maximum value, and the peak or the center-of-mass of the top-ranked cluster was selected as the stimulation site. The left stimulation site was warped to MNI space, reflected in the x-direction (left–right) and warped back to native space to identify the homolog right-hemispheric site for cathodal-tDCS.

### Delayed word reading task in MEG

Each trial began with a fixation cross displayed for 3200–3500 ms (pre-stimulus period), which was followed by a word that was presented for 750 ms. The word was replaced by a blank screen for 3500 ms (delay period), followed by a speech bubble which stayed on the screen for 4000 ms (Fig. 2B). Participants were instructed to withhold saying the word out loud until the speech prompt appeared. The stimuli were projected onto the screen via mirrors from an LCD projector placed outside the magnetically-shielded room. The experiment was implemented using Presentation (Neurobehavioral Systems, Inc., Berkeley, CA).

SOS, the stochastic optimization algorithm^65^, was employed for generating 7 non-overlapping word lists matched for frequency, number of syllables, word length and bigram frequency. Each word-list consisted of 200 words, split into 5 runs of 40 trials. The lists were counterbalanced across pre-/post-tDCS sessions and participants. The reaction time and accuracy were scored by an experimenter blinded to the HD-tDCS polarity; see Supplementary information for details on how these performance measures were scored.

In the current paper, we focused on the effects of tDCS during the pre-stimulus or fixation period (pseudo resting-state data as carried out in our previous work^23^ and the effects during the delay period (when participants were holding the word in their minds).

### MEG analysis of the pre-stimulus and induced oscillatory activity with HD-TDCS

The MEG signals from the fixation/pre-stimulus and the delay/post-stimulus time windows from the delayed word reading task were subjected to relative power and MSE computations, using the same steps as with the rsMEG data. The estimates extracted from the voxels within the stimulation sites were analyzed using linear mixed models in R^66^. Separate models were generated for comparisons of delta, theta, alpha, beta, and low-gamma power, and mean MSE in coarse, fine and intermediate scale ranges. For all linear mixed models, independent variables were contrast-coded and dependent variables were rank-transformed. For significant results, we report marginal and conditional R^2^, a measure of explained variances in the model, as a global measure of effect sizescomputed using methods discussed^67^ in Nakagawa et al.^67^ and as implemented in sjPlot package in R. Custom contrasts, as informed by the linear mixed models, were generated and used with emmeans package in R for posthoc evaluations of significant 2- and 3-way interaction findings. The figures were generated using ggplot2^68^ in R or Microsoft Office Suite products such as Excel or PowerPoint.

We conducted between-group analysis to demonstrate pathological oscillatory slowing and alterations in signal complexity in the sites selected for stimulation within the perilesional cortex, compared to the same areas in healthy controls. Mean data across all pre-stimulation sites in patients was compared with controls. Importantly as noted earlier, the left site for anodal-tDCS was localized based on peak perilesional abnormalities between patient and control groups. The right site for cathodal-tDCS was then selected as a homolog of the left site. Based on this, it was unclear how the MSE and the relative power estimates would in the right hemisphere stimulation sites and the direction of the differences (i.e., increases or decreases) in stroke patients compared to the controls. For between-group analysis, the focus is therefore on differential effects related to the site of stimulation (left vs. right) and groups (stroke vs. controls), with a particular interest in the estimates at the right stimulation site (Fig. 4A). The model for this analysis included fixed effects and interactions of group (stroke, controls), site (left, right stimulation site) and segment or time windows (pre-stimulus, delay). By-subject random-intercepts and varying slopes defined by the fixed effect *site* were included. See Supplementary Table 4 for the model setup and R code snippet.

Before assessing the effects of tDCS, we were interested in whether pathological MEG activity in perilesional areas was associated with language dysfunction. We ran Spearman’s rank correlation analysis between language impairment severity scores (WAB-AQ) and MSE as well as power spectral measures extracted from individual left stimulation sites. Informed by the between-group differences, we also interrogated relationships between language severity scores and MSE and power measures extracted from the right stimulation sites.

To assess the effects of tDCS within stroke patients, particularly reduction of oscillatory abnormalities, the model included the fixed effects and interactions of stimulation site, segment, stimulation conditions (sham, anodal-tDCS, cathodal-tDCS) and the during-stimulation version of the repetition task (Easy, Hard). We included the version of the training task in this analysis because our behavioral results indicated significant improvement on the post-tDCS repetition task in patients receiving the Hard battery but not in those who received the Easy battery. In order to account for such behavioral differences, the task version was included in our analysis of tDCS effects on MEG measures. A difference, post minus pre, in values within stimulation conditions was entered as the dependent variable. By-subject random-intercepts and varying slopes defined by the *stimulation condition* fixed effect were included. See Supplementary Table 5 for the model setup and R code snippet.

Finally, we assessed correlations between changes in spectral and MSE measures with tDCS and improvements on the post-tDCS assessment of repetition performance using Spearman’s rank correlations.

## Supplementary information

Supplementary Information
