# Supplementary material for: High definition transcranial direct current stimulation modulates abnormal neurophysiological activity in post-stroke aphasia
Source: Sci Rep. 2020 Nov 12;10:19625. doi: 10.1038/s41598-020-76533-0 (PMC7665190; doi:10.1038/s41598-020-76533-0)
Supplement: Supplementary file 1 — Supplementary Information [file 41598_2020_76533_MOESM1_ESM.docx]

**High definition transcranial direct current stimulation modulates abnormal neurophysiological activity in post-stroke aphasia**

Priyanka P. Shah-Basak^1,2*^, Gayatri Sivaratnam,^1^ Selina Teti,^1^ Alexander Francois-Nienaber,^1^ Maryam Yossofzai,^1^ Sabrina Armstrong,^1^ Sumiti Nayar,^6^ Regina Jokel,^1,2,4^ Jed Meltzer^1,2,3,5^

1. Rotman Research Institute, Baycrest Health Sciences Centre, Toronto, ON, M6A 2E1 Canada

2. Canadian Partnership for Stroke Recovery, Ottawa, ON, K1G 5Z3 Canada

3. Department of Speech-Language Pathology, University of Toronto, Toronto, ON, M5S 1A1 Canada

4. Department of Speech-Language Pathology, Baycrest Health Sciences, Toronto, ON, M6A 2E1 Canada

5. Department of Psychology, University of Toronto, Toronto, ON, M5S 1A1 Canada

6. Department of Medicine, McMaster University, Hamilton, ON L8S 4L8 Canada

**Corresponding author:**

**Priyanka P. Shah-Basak, PhD**

Department of Neurology, Medical College of Wisconsin

8701 W. Watertown Plank Rd, Wauwatosa, WI 53226 USA

prishah@mcw.edu

| Abbreviated title: | | Neurophysiological effects of HD-tDCS in stroke aphasia |
| --- | --- | --- |
| Figures: | | 5 |
| Tables: | | 2 |
| Supplementary material: | |  |
|  | Figures  Tables | 3  6 |
|  | |  |

*Note that the current address of the first author (Shah-Basak) is Department of Neurology, Medical College of Wisconsin, Milwaukee, WI 53226 USA

**SUPPLEMENTARY MATERIAL**

**METHODS**

**Sentence repetition exercises**

The same version of the task, i.e. Easy or Hard, was used during the baseline, and for training and post-tDCS assessments but the stimulus sets used for training were different from baseline and post-tDCS assessments.

A four-step procedure, described in Marangolo et al. (2016), was used during tDCS to progressively help the patient to repeat the sentences or phrases correctly. Step 1: the experimenter presented the full sentence or part of the sentence (depending on the version) and asked the patient to repeat it. If the patient was successful then the experimenter moved on to the next stimulus within that set. If the patient could not repeat or made errors, the experimenter moved on to the next step. Step 2 - 4: For the Easy version, the experimenter presented the segment “with a pause between syllables, prolonged the vowel sound, exaggerated the articulatory gestures and asked the patient to do the same.” For the Hard version, the experimenter divided the sentence into halves or thirds and asked the patient to repeat the sentence parts. At any point during these steps, if the patient was able to successfully repeat, the experimenter would ask them to repeat it again without any help. If they are successful, then the experimenter would move to the next stimulus. If however after four trials, the patient is still unable to repeat the full segment, the experimenter would move to the next stimulus and repeat the steps.

**Baseline and post-tDCS scoring of the sentence repetition task**

For the Easy version, scoring was based on each individual phrase repeated successfully. Accuracy was computed as a proportion of the number of verbatim recalls by the total number of words in the original sentence/phrase. For the Hard version, the number of verbatim words recalled in each sentence/segment was scored. Accuracy was computed as a proportion of verbatim recalls by the total number of words in the original sentence. Extra points were given based on the number of gist words/phrases. Gist words/phrases were substitutions of the original word/phrase without changing the main idea or the meaning of the sentence; in these cases, the transcribed words did not exactly match the target words. Along with accuracy, the scorer also noted the type of errors the patients made. The errors were categorized as phonological, syntactical, semantic, mixed or no responses.

For the Hard version, the gist type was also noted as substitutions that were semantic, syntactic or both. An example of gist words/phrases with semantic and syntactic substitution is as follows‒a patient substituted: “she wanted to go with a friend but no one was free” to “she wanted to be with a friend but he was not available.” The total score was calculated by taking the sum of the number of verbatim words recalled and the number of gist points (Meltzer et al., 2015; Meltzer et al., 2017).

**Delayed word reading task in MEG**

**Word stimuli selection and word list generation for multiple MEG sessions**

The stimuli consisted of nouns between 3-10 characters in length extracted from the SUBTLEX-US corpus (Brysbaert & New, 2009; Van Heuven et al., 2014). From this list, words that were lemmas (e.g. “car” but not “cars”) were filtered using the WordNetLemmatizer function in Python’s Natural Language Toolkit’s (Bird et al., 2009). Next, all 1- and 3-syllable words and words with a Zipf value (see below) greater than 3.15 and less than 6.29 were extracted. Two experimenters manually went through this list to remove words that were deemed to have negative connotations (e.g., prostitute, murderer) or that were otherwise considered inappropriate (e.g., snot, idiot), which resulted in a final population of 1942 words. Orthographic norms such as bigram frequency, word length and neighborhood count were obtained from the N-WATCH corpus (Davis, 2005) for the final list to supplement the norms included in the SUBTLEX-US corpus.

**Generating matched lists for multiple instances of MEG experiment**

We used SOS, the stochastic optimization algorithm and software package available freely for MATLAB (Armstrong, Watson, Plaut, 2012; [http://sos.cnbc.cmu.edu](http://sos.cnbc.cmu.edu/)) for generating 7 non-overlapping lists matched for word frequency, number of syllables, word length and bigram frequency. SOS was used first to generate 4 subpopulations consisting of low and high frequency words with either 1 or 3 syllables. The categorization of words by word frequency was done based on the Zipf value. Zipf value between 3 and 4 is considered the “tipping point” from low to high word frequency (Van Heuven et al., 2014). Words with a Zipf value greater than 3.65 was classified as high frequency and those equal to or below 3.65 were classified as low frequency words. Words from the 4 subpopulations were then pooled using a second SOS script to generate 7 lists that consisted of 200 words each, 50 words per condition – low-frequency, 1 syllable (low1), low-frequency, 3 syllables (low3), high-frequency, 1 syllable (high1) and high-frequency, 3 syllable (high3). These lists were also matched for bigram frequency and word length. Finally, to allow participants to have rest periods inside the MEG, each list was split into 5 runs of 40 trials, each run with 10 words from each condition, which were matched for bigram frequency and word length. The order of lists was counterbalanced across participants for their use during the pre/post-tDCS MEG sessions.

**Scoring**

The onset of the response was measured from the speech prompt to the time that the participant started speaking, excluding any unintentional sounds such as heavy breathing or coughing. Correct responses were scored based on clear, intelligible responses. Semantic and phonological paraphasias were scored as incorrect, as were unintelligible responses, such as a mumble, and trials with no response within the allotted time window.

**RESULTS**

**Behavioural Results: Delayed word reading task performance**

The main effects of lexical variables: word frequency (F(1, 9) = 13.9, p = 0.005) and number of syllables (F(1, 9) = 16.7, p = 0.003) were significant on accuracy but not on reaction time; the accuracy was lower for the 3-syllable and for the low-frequency words (Supplementary Figure 1). The effects of lexical variables on performance were not significant in controls.

**MEG results**

**Pre-stimulus and induced oscillatory and MSE differences between groups**


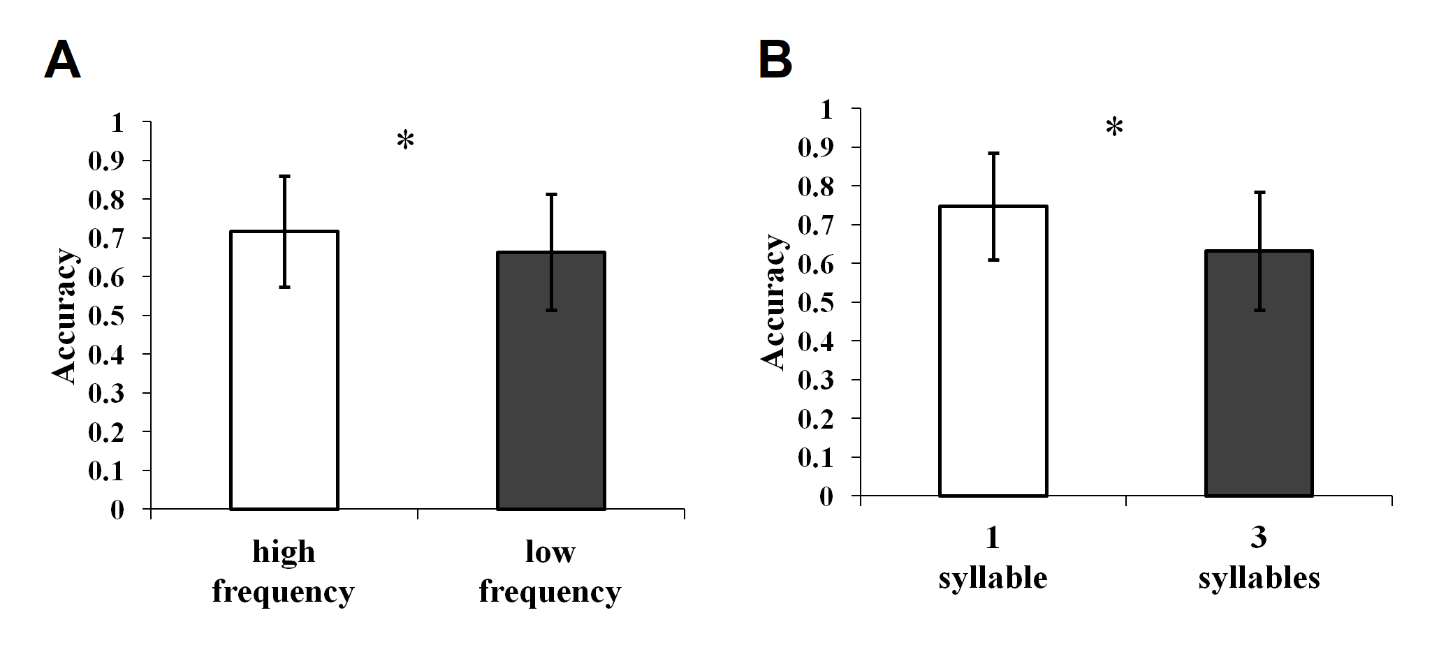


**Supplementary Figure 1.** Accuracy differences in stroke patients on the delayed word reading task based on lexical variables; **(A)** word frequency and **(B)** number of syllables. Error bars indicate SE and asterisks (*) indicate statistical significance at p<0.05.

The effects related to segment (pre-stimulus, delay) are reported here as supplementary information. The group×segment interaction (t=2.2, p=0.031) and the fixed effect of site (t=2.5, p=0.021) were significant for the intermediate-scale MSE, indicating that it was higher in the right than in the left site across both groups, and it increased during the delay period in patients across both sites (Supplementary Figure 2A). For low-gamma, the group×segment (t=2.2, p=0.030) interaction was also significant, indicating that the low-gamma power was reduced during the delay compared to the pre-stimulus period in patients (Supplementary Figure 2B).


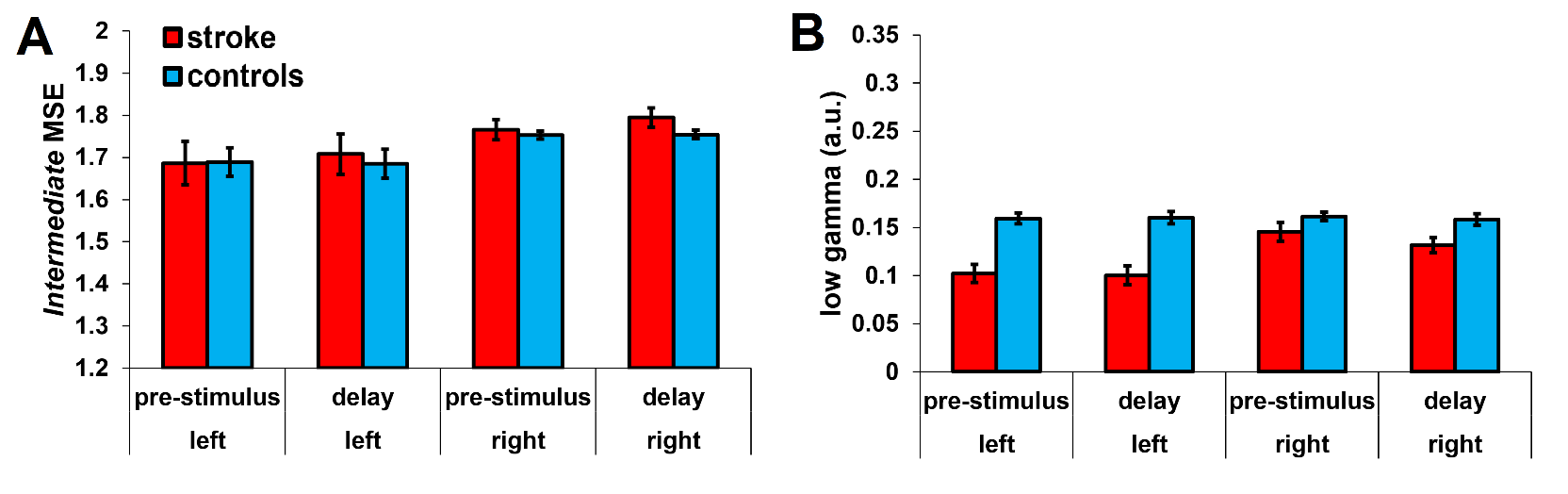


**Supplementary Figure 2.** Oscillatory and multi-scale entropy (MSE) differences in the left vs. the right hemispheric stimulation sites during the pre-stimulus and delay period of the delayed word reading task. Group (stroke, controls) by segment (prestimulus, delay) interactions were significant for intermediate-scale MSE (A) and low-gamma (B).


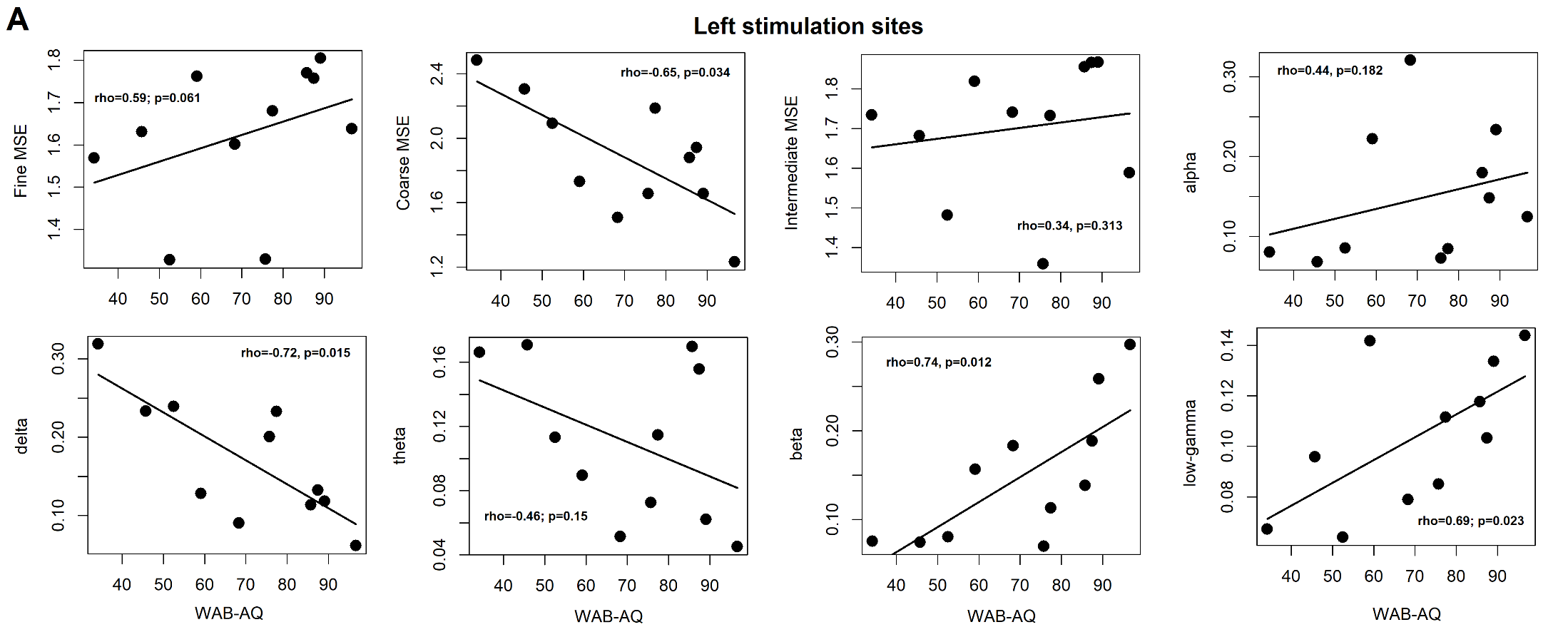


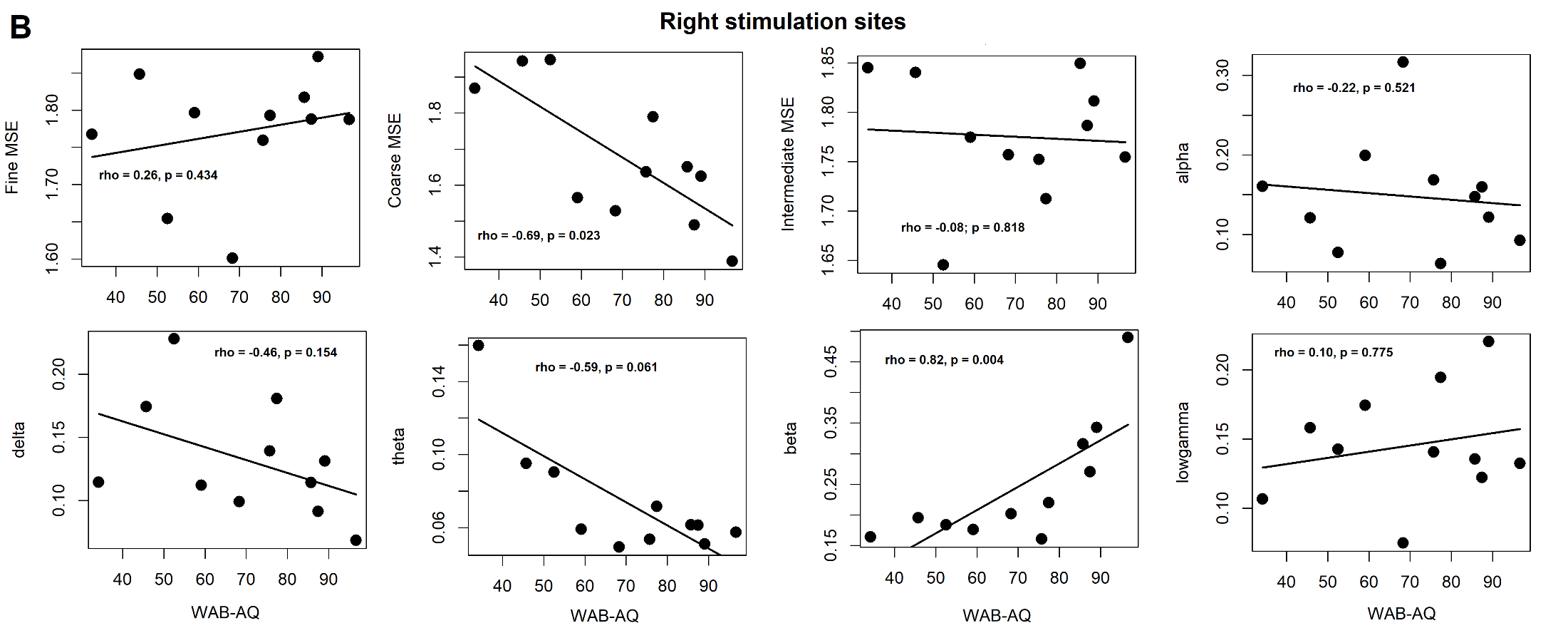


**Supplementary Figure 3.** Scatter plots demonstrating a relationship between MEG measures (MSE and spectral measures) and aphasia severity scores. Relationships for measures derived from the **(A)** left/perilesional stimulation site and the **(B)** right/contralateral stimulation site. WAB-AQ = Western Aphasia Battery – Aphasia Quotient; MSE = multiscale entropy

| **Supplementary Table 1. Demographics of the healthy controls** | | | |
| --- | --- | --- | --- |
| **ID** | **Age (y)** | **Education (y)** | **Sex** |
| C1 | 69 | 15 | M |
| C2 | 75 | 19 | M |
| C3 | 69 | 17 | M |
| C4 | 49 | 16 | M |
| C5 | 71 | 18 | M |
| C6 | 61 | 17 | M |
| C7 | 64 | 16 | F |
| C8 | 71 | 20 | F |
| C9 | 44 | 16 | F |
| C10 | 31 | 19 | F |
| **Mean**  **(SD)** | **60.4**  **(14.4)** | **17.3**  **(1.6)** | **6M** |

| **Supplementary Table 2. Two versions of the sentence repetition exercises designed to ensure engagement of patients with varying degrees of language severity. Example sentences used in the experiment** | | |
| --- | --- | --- |
| **Versions** |  | **Example sentences** |
| Easy |  | Car  That car  That big car  That big car drives  That big car drives well  That big car sometimes drives well |
| Hard |  | I often watch television after work.  Do you like to watch the news?  I like channel 3 but my wife watches channel 10.  Do you think television is a waste of time? Watching a movie is easier than reading a book. |

**Supplementary Table 3.** Accuracy in the phrase/sentence repetition task in individual patients.

|  | **Task version** | **Anodal-tDCS** | **Cathodal-tDCS** | **Sham-tDCS** | **Baseline** |
| --- | --- | --- | --- | --- | --- |
| **P01** | Easy | 0.839 | 0.953 | 0.918 | 0.837 |
| **P03** | Easy | 0.608 | 0.574 | 0.615 | 0.628 |
| **P04** | Easy | 0.691 | 0.584 | 0.784 | 0.747 |
| **P09** | Easy | 0.000 | 0.000 | 0.000 | 0.000 |
| **P10** | Easy | 0.452 | 0.611 | 0.375 | 0.540 |
| **P02** | Hard | 0.227 | 0.164 | 0.202 | 0.187 |
| **P05** | Hard | 0.845 | 0.672 | 0.673 | 0.683 |
| **P06** | Hard | 0.629 | 0.663 | 0.358 | 0.575 |
| **P07** | Hard | 0.826 | 0.817 | 0.692 | 0.740 |
| **P08** | Hard | 0.760 | 0.826 | 0.552 | 0.668 |
| **P11** | Hard | 0.386 | 0.397 | 0.352 | 0.306 |

| Supplementary Table 4. R code snippet and linear mixed model output for the between-group analysis on the differences in oscillatory and MSE values during the pre-stimulus and delay time windows in the MEG delayed word reading task. | | | | | | | | | | | | |
| --- | --- | --- | --- | --- | --- | --- | --- | --- | --- | --- | --- | --- |
| **R code snippet:**  mat<-rbind(c(1,-1))  cmat<-ginv(mat)  summary(mod1<-lmer(rank(mean_pow_mse)~group*site*segment+(1+site\|subjid:group)  , contrasts = list(group = cmat, site=cmat, segment=cmat)))  where mean_pow_mse = mean power or MSE values; group (stroke, controls); stimulation site (left, right); segment(delay, fixation) | | | | | | | | | | | | |
|  | **Mean fine-scale MSE (4-10)** | | | | | | | | | | | |
| *Predictors* | *Estimates* | | | *t-statistic* | | | *p* | | | | *df* | |
| **group** | **-17.30** | | | **-2.14** | | | **0.033** | | | | **20.00** | |
| group:segment | 2.73 | | | 1.24 | | | 0.215 | | | | 40.00 | |
| segment | -1.16 | | | -1.05 | | | 0.292 | | | | 40.00 | |
| **site** | **-22.27** | | | **-5.17** | | | **<0.001** | | | | **20.00** | |
| ***group:site*** | ***-14.14*** | | | ***-1.64*** | | | ***0.101*** | | | | ***20.00*** | |
| site:segment | 4.32 | | | 1.96 | | | 0.049 | | | | 40.00 | |
| group:site:segment | -1.45 | | | -0.33 | | | 0.741 | | | | 40.00 | |
|  | | | **Mean intermediate-scale MSE (11-20)** | | | | | | | | | |
| *Predictors* | | | *Estimates* | | | *t-statistic* | | | *p* | *df* | | |
| group | | | 12.09 | | | 1.38 | | | 0.168 | 20.00 | | |
| group:segment | | | 6.91 | | | 2.24 | | | 0.025 | 40.00 | | |
| segment | | | 3.18 | | | 2.06 | | | 0.039 | 40.00 | | |
| site | | | -13.55 | | | -2.50 | | | 0.012 | 20.00 | | |
| ***group:site*** | | | ***-1.82*** | | | ***-0.17*** | | | ***0.867*** | ***20.00*** | | |
| site:segment | | | -4.36 | | | -1.42 | | | 0.157 | 40.00 | | |
| group:site:segment | | | -2.91 | | | -0.47 | | | 0.637 | 40.00 | | |
|  | | **Mean coarse-scale MSE (21-60)** | | | | | | | | | | |
| *Predictors* | | *Estimates* | | | *t-statistic* | | | *p* | | | | *df* |
| group | | 30.32 | | | 3.75 | | | <0.001 | | | | 20.00 |
| group:segment | | 2.36 | | | 1.22 | | | 0.223 | | | | 40.00 |
| segment | | 1.86 | | | 1.92 | | | 0.055 | | | | 40.00 |
| site | | 0.32 | | | 0.09 | | | 0.928 | | | | 20.00 |
| ***group:site*** | | ***16.00*** | | | ***2.28*** | | | ***0.023*** | | | | ***20.00*** |
| site:segment | | -2.55 | | | -1.31 | | | 0.190 | | | | 40.00 |
| group:site:segment | | -5.64 | | | -1.45 | | | 0.146 | | | | 40.00 |
|  | | | **Delta power** | | | | | | | | | |
| *Predictors* | | | *Estimates* | | | *t-statistic* | | | *p* | *df* | | |
| **group** | | | **28.36** | | | **3.40** | | | **0.001** | **20.00** | | |
| group:segment | | | 2.73 | | | 0.79 | | | 0.429 | 40.00 | | |
| segment | | | 2.00 | | | 1.16 | | | 0.246 | 40.00 | | |
| site | | | 3.02 | | | 0.91 | | | 0.362 | 20.00 | | |
| ***group:site*** | | | ***10.14*** | | | ***1.53*** | | | ***0.126*** | ***20.00*** | | |
| site:segment | | | -0.95 | | | -0.28 | | | 0.782 | 40.00 | | |
| group:site:segment | | | -6.45 | | | -0.94 | | | 0.349 | 40.00 | | |
|  | | | **Theta power** | | | | | | | | | |
| *Predictors* | | | *Estimates* | | | *t-statistic* | | | *p* | *df* | | |
| group | | | 17.57 | | | 1.87 | | | 0.062 | 20.00 | | |
| group:segment | | | 1.45 | | | 0.56 | | | 0.576 | 40.00 | | |
| segment | | | 0.07 | | | 0.05 | | | 0.958 | 40.00 | | |
| site | | | 2.98 | | | 0.81 | | | 0.419 | 20.00 | | |
| ***group:site*** | | | ***24.73*** | | | ***3.36*** | | | ***0.001*** | ***20.00*** | | |
| site:segment | | | -1.73 | | | -0.66 | | | 0.507 | 40.00 | | |
| group:site:segment | | | -3.73 | | | -0.72 | | | 0.474 | 40.00 | | |
|  | | **Alpha power** | | | | | | | | | | |
| *Predictors* | | *Estimates* | | | *t-statistic* | | | *p* | | | | *df* |
| group | | -3.82 | | | -0.39 | | | 0.693 | | | | 19.99 |
| group:segment | | -0.27 | | | -0.15 | | | 0.884 | | | | 40.00 |
| segment | | 4.95 | | | 5.29 | | | <0.001 | | | | 40.00 |
| site | | -6.80 | | | -1.27 | | | 0.203 | | | | 20.00 |
| ***group:site*** | | ***0.32*** | | | ***0.03*** | | | ***0.976*** | | | | ***20.00*** |
| site:segment | | -7.32 | | | -3.91 | | | <0.001 | | | | 40.00 |
| group:site:segment | | 5.55 | | | 1.48 | | | 0.139 | | | | 40.00 |
|  | | **Beta power** | | | | | | | | | | |
| *Predictors* | | *Estimates* | | | *t-statistic* | | | *p* | | | | *df* |
| group | | -27.23 | | | -3.27 | | | 0.001 | | | | 20.00 |
| group:segment | | -1.18 | | | -0.68 | | | 0.499 | | | | 40.00 |
| segment | | -2.55 | | | -2.91 | | | 0.004 | | | | 40.00 |
| site | | -14.14 | | | -5.46 | | | <0.001 | | | | 20.00 |
| ***group:site*** | | ***-17.82*** | | | ***-3.44*** | | | ***0.001*** | | | | ***20.00*** |
| site:segment | | 2.64 | | | 1.51 | | | 0.131 | | | | 40.00 |
| group:site:segment | | 3.64 | | | 1.04 | | | 0.298 | | | | 40.00 |
|  | | **Low-gamma power** | | | | | | | | | | |
| *Predictors* | | *Estimates* | | | *t-statistic* | | | *p* | | | | *df* |
| group | | -29.84 | | | -4.12 | | | <0.001 | | | | 20.00 |
| group:segment | | -5.45 | | | -2.24 | | | 0.025 | | | | 40.00 |
| segment | | -4.20 | | | -3.46 | | | 0.001 | | | | 40.00 |
| site | | -11.52 | | | -3.19 | | | 0.001 | | | | 20.00 |
| ***group:site*** | | ***-24.00*** | | | ***-3.32*** | | | ***0.001*** | | | | ***20.00*** |
| site:segment | | 8.09 | | | 3.33 | | | 0.001 | | | | 40.00 |
| group:site:segment | | 7.91 | | | 1.63 | | | 0.104 | | | | 40.00 |

| **Supplementary Table 5. R code snippet and linear mixed model outputs for the within-stroke patients’ analyses for the effects of HD-tDCS on spectral and MSE measures** | | | | | | | | |
| --- | --- | --- | --- | --- | --- | --- | --- | --- |
| **R code snippet:**  n <- 3  M <- matrix(0,nrow=n,ncol=n*(n-1)/2)  comb <- combn(n,2)  M[cbind(comb[1,],1:(n*(n-1)/2))] <- -1  M[cbind(comb[2,],1:(n*(n-1)/2))] <- 1  cmat1<-ginv(t(M[,1:2]))  summary(lmmod1<-lmer(rank(diff_pow_mse) ~ stimtype*site*segment* sentrep  + (1+stimtype\|subjid)  ,data=lcdiff  ,contrasts = list(sentrep = cmat, site =cmat, segment=cmat, stimtype=cmat1)))  where diff_pow_mse = post- vs. pre-tDCS differences in power or MSE values; stimtype (sham, anodal, cathodal), stimulation site (left, right); segment (delay, fixation); sentrep (Easy, Hard)  stimtype1 = anodal-tDCS compared to sham and cathodal-tDCS  stimtype2 = cathodal-tDCS compared to sham and anodal-tDCS | | | | | | | | |
| **Difference post- vs. pre-tDCS** | **Theta power** | | | | | | | |
| *Predictors* | *Estimates* | | *t-statistic* | | | *p* | *df* | |
| site1:segment1:sentrep1 | 12.50 | | 0.57 | | | 0.567 | 90.00 | |
| site1:sentrep1 | 5.87 | | 0.54 | | | 0.591 | 90.00 | |
| stimtype1:site1:segment1:sentrep1 | -55.67 | | -1.04 | | | 0.298 | 90.00 | |
| ***stimtype1:site1*** | ***40.09*** | | ***3.00*** | | | ***0.003*** | ***90.00*** | |
| stimtype2:site1:segment1:sentrep1 | 45.92 | | 0.86 | | | 0.391 | 90.00 | |
| site1:segment1 | -9.92 | | -0.91 | | | 0.364 | 90.00 | |
| stimtype1:segment1 | 8.38 | | 0.63 | | | 0.531 | 90.00 | |
| sentrep1 | 5.96 | | 0.48 | | | 0.634 | 9.02 | |
| stimtype1:site1:sentrep1 | 14.52 | | 0.54 | | | 0.588 | 90.00 | |
| segment1 | -2.76 | | -0.50 | | | 0.614 | 90.00 | |
| stimtype2:sentrep1 | 38.13 | | 1.93 | | | 0.054 | 9.25 | |
| stimtype1:segment1:sentrep1 | -4.07 | | -0.15 | | | 0.879 | 90.00 | |
| stimtype2:segment1 | 4.96 | | 0.37 | | | 0.711 | 90.00 | |
| stimtype1 | 5.02 | | 0.49 | | | 0.626 | 9.60 | |
| stimtype2:site1:sentrep1 | -6.69 | | -0.25 | | | 0.803 | 90.00 | |
| site1 | 1.77 | | 0.32 | | | 0.746 | 90.00 | |
| stimtype1:site1:segment1 | 25.33 | | 0.95 | | | 0.344 | 90.00 | |
| segment1:sentrep1 | 1.71 | | 0.16 | | | 0.876 | 90.00 | |
| stimtype2:site1 | 10.45 | | 0.78 | | | 0.435 | 90.00 | |
| stimtype2 | 9.84 | | 1.00 | | | 0.320 | 9.25 | |
| stimtype2:segment1:sentrep1 | -1.32 | | -0.05 | | | 0.961 | 90.00 | |
| stimtype1:sentrep1 | -5.29 | | -0.26 | | | 0.797 | 9.60 | |
| stimtype2:site1:segment1 | 3.04 | | 0.11 | | | 0.910 | 90.00 | |
| **Difference post- vs. pre-tDCS** | | **Mean coarse-scale MSE (21-60)** | | | | | | |
| *Predictors* | | *Estimates* | | *t-statistic* | *p* | | | *df* |
| site1:segment1:sentrep1 | | 7.79 | | 0.36 | 0.717 | | | 90.00 |
| site1:sentrep1 | | 5.64 | | 0.52 | 0.600 | | | 90.00 |
| stimtype1:site1:segment1:sentrep1 | | -48.97 | | -0.93 | 0.353 | | | 90.00 |
| ***stimtype1:site1*** | | ***33.79*** | | ***2.57*** | ***0.010*** | | | ***90.00*** |
| stimtype2:site1:segment1:sentrep1 | | 1.23 | | 0.02 | 0.981 | | | 90.00 |
| site1:segment1 | | 0.84 | | 0.08 | 0.938 | | | 90.00 |
| stimtype1:segment1 | | 3.09 | | 0.23 | 0.814 | | | 90.00 |
| sentrep1 | | -6.45 | | -0.48 | 0.635 | | | 9.00 |
| stimtype1:site1:sentrep1 | | -3.58 | | -0.14 | 0.892 | | | 90.00 |
| segment1 | | -1.25 | | -0.23 | 0.817 | | | 90.00 |
| stimtype2:sentrep1 | | 3.98 | | 0.26 | 0.797 | | | 13.86 |
| stimtype1:segment1:sentrep1 | | -14.98 | | -0.57 | 0.570 | | | 90.00 |
| stimtype2:segment1 | | -10.86 | | -0.82 | 0.410 | | | 90.00 |
| stimtype1 | | 1.47 | | 0.12 | 0.901 | | | 9.00 |
| stimtype2:site1:sentrep1 | | -29.75 | | -1.13 | 0.259 | | | 90.00 |
| site1 | | 9.35 | | 1.74 | 0.082 | | | 90.00 |
| stimtype1:site1:segment1 | | 19.68 | | 0.75 | 0.455 | | | 90.00 |
| segment1:sentrep1 | | 2.56 | | 0.24 | 0.812 | | | 90.00 |
| stimtype2:site1 | | 13.38 | | 1.02 | 0.310 | | | 90.00 |
| stimtype2 | | 9.86 | | 1.28 | 0.202 | | | 13.86 |
| stimtype2:segment1:sentrep1 | | -20.88 | | -0.79 | 0.428 | | | 90.00 |
| stimtype1:sentrep1 | | -26.14 | | -1.10 | 0.270 | | | 9.00 |
| stimtype2:site1:segment1 | | -3.22 | | -0.12 | 0.903 | | | 90.00 |
|  | | | | | | | | |
| **Difference post- vs. pre-tDCS** | **Mean fine-scale MSE (4-10)** | | | | | | | |
| *Predictors* | *Estimates* | | *t-statistic* | | | *p* | *df* | |
| site1:segment1:sentrep1 | 1.29 | | 0.07 | | | 0.941 | 81.00 | |
| site1:sentrep1 | -19.91 | | -2.29 | | | 0.022 | 81.00 | |
| stimtype1:site1:segment1:sentrep1 | -76.48 | | -1.79 | | | 0.073 | 81.00 | |
| stimtype1:site1 | 26.20 | | 2.46 | | | 0.014 | 81.00 | |
| stimtype2:site1:segment1:sentrep1 | 8.97 | | 0.21 | | | 0.834 | 81.00 | |
| site1:segment1 | 4.79 | | 0.55 | | | 0.583 | 81.00 | |
| stimtype1:segment1 | -6.41 | | -0.60 | | | 0.548 | 81.00 | |
| sentrep1 | 13.31 | | 1.28 | | | 0.200 | 9.00 | |
| ***stimtype1:site1:sentrep1*** | ***-54.01*** | | ***-2.53*** | | | ***0.011*** | ***81.00*** | |
| segment1 | -10.67 | | -2.45 | | | 0.014 | 81.00 | |
| stimtype2:sentrep1 | 13.43 | | 0.48 | | | 0.631 | 9.00 | |
| stimtype1:segment1:sentrep1 | 4.43 | | 0.21 | | | 0.836 | 81.00 | |
| stimtype2:segment1 | -12.49 | | -1.17 | | | 0.242 | 81.00 | |
| stimtype1 | 5.25 | | 0.33 | | | 0.742 | 9.00 | |
| stimtype2:site1:sentrep1 | -21.62 | | -1.01 | | | 0.311 | 81.00 | |
| site1 | 11.67 | | 2.68 | | | 0.007 | 81.00 | |
| stimtype1:site1:segment1 | -12.16 | | -0.57 | | | 0.569 | 81.00 | |
| segment1:sentrep1 | -17.36 | | -1.99 | | | 0.046 | 81.00 | |
| ***stimtype2:site1*** | ***24.86*** | | ***2.33*** | | | ***0.020*** | ***81.00*** | |
| stimtype2 | 9.21 | | 0.66 | | | 0.509 | 9.00 | |
| stimtype2:segment1:sentrep1 | -29.32 | | -1.37 | | | 0.170 | 81.00 | |
| stimtype1:sentrep1 | -8.70 | | -0.27 | | | 0.785 | 9.00 | |
| stimtype2:site1:segment1 | 20.82 | | 0.98 | | | 0.329 | 81.00 | |
| **Difference post- vs. pre-tDCS** | **Low-gamma power^#^** | | | | | | | |
| *Predictors* | *Estimates* | | *t-statistic* | | | *p* | *df* | |
| site1:segment1:sentrep1 | -4.75 | | -0.27 | | | 0.784 | 99.00 | |
| site1:sentrep1 | -23.75 | | -2.74 | | | **0.006** | 99.00 | |
| stimtype1:site1:segment1:sentrep1 | 27.43 | | 0.65 | | | 0.518 | 99.00 | |
| stimtype1:site1 | 9.28 | | 0.88 | | | 0.382 | 99.00 | |
| stimtype2:site1:segment1:sentrep1 | 11.62 | | 0.27 | | | 0.784 | 99.00 | |
| site1:segment1 | -6.12 | | -0.71 | | | 0.479 | 99.00 | |
| stimtype1:segment1 | 1.50 | | 0.14 | | | 0.888 | 99.00 | |
| sentrep1 | 6.34 | | 0.61 | | | 0.540 | 11.00 | |
| ***stimtype1:site1:sentrep1*** | ***-51.77*** | | ***-2.44*** | | | ***0.015*** | ***99.00*** | |
| segment1 | 4.36 | | 1.01 | | | 0.314 | 99.00 | |
| stimtype2:sentrep1 | 23.94 | | 1.07 | | | 0.284 | 11.00 | |
| stimtype1:segment1:sentrep1 | -6.00 | | -0.28 | | | 0.777 | 99.00 | |
| stimtype2:segment1 | 7.67 | | 0.72 | | | 0.470 | 99.00 | |
| stimtype1 | 14.07 | | 0.98 | | | 0.329 | 11.00 | |
| stimtype2:site1:sentrep1 | -8.78 | | -0.41 | | | 0.679 | 99.00 | |
| site1 | 5.56 | | 1.28 | | | 0.199 | 99.00 | |
| stimtype1:site1:segment1 | 1.88 | | 0.09 | | | 0.929 | 99.00 | |
| segment1:sentrep1 | 1.52 | | 0.18 | | | 0.861 | 99.00 | |
| stimtype2:site1 | 6.99 | | 0.66 | | | 0.510 | 99.00 | |
| stimtype2 | 5.53 | | 0.50 | | | 0.621 | 11.00 | |
| stimtype2:segment1:sentrep1 | -4.74 | | -0.22 | | | 0.823 | 99.00 | |
| stimtype1:sentrep1 | 40.56 | | 1.41 | | | 0.160 | 11.00 | |
| stimtype2:site1:segment1 | -1.61 | | -0.08 | | | 0.940 | 99.00 | |

^#^this model converged using restricted maximum likelihood method (REML=TRUE) and using a ‘bobyqa’ optimization algorithm.

**Supplementary Table 6.** Changes in spectral and MSE measures with tDCS in individual patients.

|  | **Anodal-tDCS** | | | | | | | | **Cathodal-tDCS** | | | | | | | | **Sham-tDCS** | | | | | | | |
| --- | --- | --- | --- | --- | --- | --- | --- | --- | --- | --- | --- | --- | --- | --- | --- | --- | --- | --- | --- | --- | --- | --- | --- | --- |
|  | **Theta** | | **Low-gamma** | | **Coarse MSE** | | **Fine MSE** | | **Theta** | | **Low-gamma** | | **Coarse MSE** | | **Fine MSE** | | **Theta** | | **Low-gamma** | | **Coarse MSE** | | **Fine MSE** | |
|  | **L** | **R** | **L** | **R** | **L** | **R** | **L** | **R** | **L** | **R** | **L** | **R** | **L** | **R** | **L** | **R** | **L** | **R** | **L** | **R** | **L** | **R** | **L** | **R** |
| **P01** | 0.013 | -0.008 | -0.011 | 0.053 | 0.081 | -0.284 | 0.004 | 0.044 | 0.001 | 0.001 | 0.009 | -0.010 | -0.058 | 0.029 | -0.010 | 0.027 | 0.003 | 0.000 | -0.004 | 0.009 | 0.000 | -0.038 | 0.007 | 0.015 |
| **P03** | 0.012 | -0.008 | 0.050 | 0.044 | -0.244 | -0.187 | 0.167 | 0.053 | 0.052 | 0.011 | 0.017 | 0.019 | -0.026 | -0.105 | 0.099 | 0.033 | -0.029 | -0.006 | 0.017 | 0.015 | -0.139 | -0.113 | 0.040 | 0.015 |
| **P04** | 0.004 | -0.007 | 0.012 | 0.009 | -0.100 | -0.084 | -0.016 | 0.017 | -0.001 | 0.001 | 0.032 | 0.015 | -0.181 | -0.084 | 0.133 | 0.025 | -0.005 | 0.000 | 0.007 | -0.005 | -0.112 | -0.093 | -0.034 | -0.037 |
| **P09** | -0.012 | 0.004 | 0.003 | 0.002 | 0.086 | -0.006 | 0.003 | -0.005 | -0.007 | 0.008 | 0.002 | -0.001 | -0.023 | 0.021 | -0.016 | 0.001 | -0.007 | -0.003 | 0.005 | 0.007 | -0.091 | -0.097 | 0.004 | 0.020 |
| **P10** | -0.014 | -0.009 | 0.024 | 0.022 | -0.103 | -0.186 | -0.004 | -0.182 | 0.013 | -0.008 | -0.009 | 0.025 | 0.158 | -0.149 | 0.106 | -0.106 | 0.003 | -0.001 | -0.014 | -0.015 | 0.026 | 0.011 | 0.013 | 0.015 |
| **P02** | 0.010 | 0.004 | 0.011 | -0.024 | -0.021 | 0.035 | 0.039 | 0.005 | -0.005 | 0.002 | 0.000 | -0.010 | -0.074 | 0.030 | 0.007 | -0.013 | 0.013 | -0.002 | -0.026 | 0.015 | 0.138 | -0.102 | -0.056 | 0.014 |
| **P05** | 0.002 | -0.001 | -0.015 | -0.009 | 0.014 | -0.026 | -0.015 | -0.043 | -0.004 | -0.001 | 0.008 | 0.017 | -0.031 | -0.091 | -0.014 | -0.015 | -0.003 | -0.001 | 0.023 | 0.039 | -0.081 | 0.022 | 0.012 | 0.063 |
| **P06** | -0.017 | -0.009 | 0.044 | 0.017 | -0.122 | -0.134 | 0.069 | -0.002 | -0.018 | -0.008 | 0.027 | 0.007 | -0.066 | -0.161 | 0.052 | -0.010 | -0.009 | -0.004 | 0.026 | -0.003 | -0.113 | -0.104 | 0.045 | -0.019 |
| **P07** | -0.002 | -0.005 | 0.007 | -0.009 | -0.023 | -0.075 | 0.005 | -0.033 | -0.009 | -0.007 | 0.001 | -0.002 | -0.088 | -0.030 | 0.007 | -0.002 | -0.024 | 0.000 | 0.024 | 0.023 | -0.144 | 0.005 | 0.033 | 0.036 |
| **P08** | -0.002 | -0.007 | 0.013 | 0.000 | -0.013 | -0.116 | 0.013 | -0.022 | 0.001 | -0.007 | 0.025 | 0.002 | 0.033 | -0.109 | 0.017 | -0.042 | -0.009 | -0.001 | -0.007 | -0.028 | -0.109 | -0.047 | -0.044 | -0.026 |
| **P11** | 0.008 | 0.004 | 0.028 | 0.009 | 0.038 | -0.016 | 0.057 | 0.014 | 0.002 | -0.001 | -0.001 | 0.006 | -0.023 | -0.031 | 0.004 | 0.017 | 0.000 | 0.000 | 0.006 | 0.002 | -0.083 | -0.086 | -0.083 | -0.089 |

L=left, perilesional site; R = right, contralateral site; shaded in grey = Hard group;
